# Advances on Electrochemiluminescent Biosensors for TB Biomarkers

**DOI:** 10.1021/acssensors.4c03517

**Published:** 2025-04-09

**Authors:** Meleskow Cox, Jaymi January, Kefilwe Vanessa Mokwebo, Sodiq T. Yussuf, Nelia Abraham Sanga, Zandile Dennis Leve, Samantha Fiona Douman, Emmanuel Iheanyichukwu Iwuoha

**Affiliations:** † SensorLab (University of the Western Cape Sensor Laboratories), Chemical Building University of the Western Cape, Bellville, 7535, Cape Town, South Africa; ‡ South African Research Chair Initiative (SARChI) Chair for NanoElectrochemistry and Sensor Technology, University of the Western Cape, Bellville, 7535, Cape Town, South Africa; § Department of Chemical Sciences, Olabisi Onabanjo University, P.M.B. 2002, Ago-Iwoye, Ogun State, Nigeria; ∥ Department of Chemistry, University of Cape Town, Rondebosch, Cape Town 7701, South Africa

**Keywords:** electrochemiluminescence, tuberculosis, biomarkers, biosensors, diagnostics, limit of detection, linear dynamic range, luminophores

## Abstract

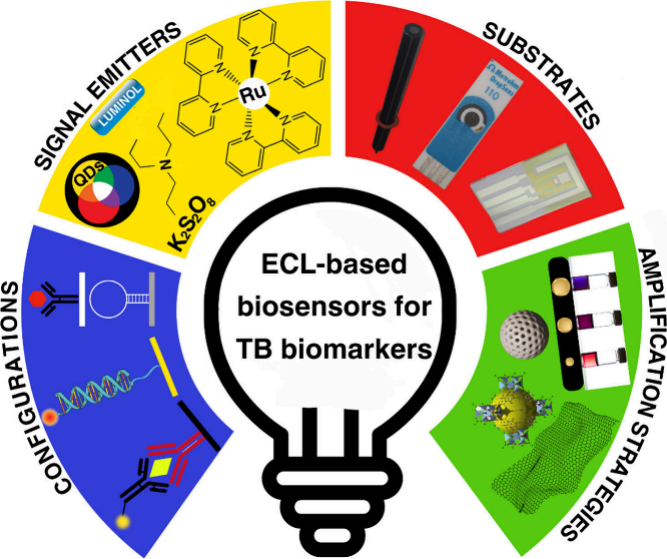

Tuberculosis (TB) is a highly contagious bacterial infection that remains a leading cause of death and persistent threat to global health. The spread of TB is exacerbated by the major limitations of conventional diagnostic approaches, such as complex technicalities, high cost, and low sensitivity. To address these challenges, recent research has focused on using electrochemiluminescence (ECL) as an alternative detection strategy coupled to biosensors. ECL biosensors leverage electrochemically generated chemiluminescence, converting electrical energy to light, as a novel transduction mechanism for TB biosensors. This unique approach offers several advantages, namely, wide linear dynamic ranges, improved device sensitivities, and prompt response times for sensitive early detection. This Review offers a comprehensive overview of advancements in ECL biosensor configurations, including detection and amplification strategies, substrates, and the development of luminophores and coreactants tailored for TB biomarker detection. The focus is on ECL biosensor designs, including biorecognition elements like immunosensors, DNA sensors, and aptasensors, along with various immobilization strategies tailored to target specific TB biomarkers. A comprehensive discussion spans biomarker detection trends over the past decade, clinical relevance, sensitivity thresholds, and detection limits. Furthermore, widely recognized TB biomarkers commonly detected in commercial diagnostic tests are discussed alongside novel markers that, while not exclusive to TB, have demonstrated clinical importance. This Review aims to highlight the potential of ECL-based biosensors as an effective means to advance an early, reliable, and accessible TB detection approach.

## Overview

The work of Koch in 1882 was fundamental to the discovery of tuberculosis (TB), which is caused by the *Mycobacterium tuberculosis* microorganism.^1,2^ TB is an airborne disease that is transmissible from one host to another.^3−5^ The disease exists largely as pulmonary TB, affecting the lungs,^6^ and is detectable and curable via treatments made available at healthcare facilities worldwide.^7^ Therefore, it is concerning that TB still ranks as a life-threatening disease in the present day. According to a series of world health organization reports (WHO),^8−10^ the estimated death rate is increasing by the day with approximately 1.4 million fatalities, and as a consequence, TB competes with incurable diseases such as diabetes for the highest death toll rate. The primary mitigation strategy to improve patient survival are early diagnosis to catch and treat the disease in those already showing symptoms.^11^ Majority of TB cases are detected using microscopy techniques as listed in Table 1. The general trends indicate that these techniques are expensive, time-consuming, complex or lack sensitivity. Hence, the available diagnostic tests have flaws that contribute to the high prevalence of TB, that hinders the effective management of the disease. A way to counter the poor management of TB would be to develop diagnostic tests that offer the following specifications, such as being portable, easy to use, high sensitivity, high specificity, ability to provide immediate results and being accessible at a low-cost, etc.^12^ In recent years, biosensors have been proposed as a diagnostic test for detecting TB. This enables early and accurate detection of TB by identifying specific biomarkers associated with the disease that improve the diagnostic sensitivity.^13^ The rapid results produced by biosensors aid in efficient treatment dispensing, which is vital for controlling the spread of the disease. Additionally, after extensive clinical trials and rigorous validations, biosensors are developed for point-of-care testing, making them well-suited for remote or resource-limited settings and are cost-effective.^14,15^ These advantages contribute to improved disease management that aids in the reduction of TB more effectively.^16^ Interestingly, electrochemiluminescence (ECL) has emerged as an exceptionally sensitive transducer for detecting TB biomarkers. Notably, Hercules^17^ and Bard et al.^18^ conducted the first in-depth investigations into ECL that has established the technique as a leading method in biosensor technology. Advancements in biosensor configurations, substrate modifications, and the development of luminophores and coreactants for detecting specific biomarkers are reviewed herein. Given that the detection is ECL-based, both labeled and label-free methods are presented. The work is arranged to present the current status in ECL biosensor research for TB biomarkers. Moreover, the review will be classified according to the TB biomarkers. Additionally, the TB clinical relevance for each biomarker will be investigated and discussed. This includes the reference to cutoff values as well as TB biomarkers detected via commercially available diagnostic tests.

**Table 1:** Advantages and Disadvantages of Commercial TB Detection Methods versus ECL

conventional methods	advantages	disadvantages	refs
culture method	gold standard	time consuming, 3–4 weeks for results	(19)
	high sensitivity	resource exhaustive	
sputum smear	easy to perform	lacks sensitivity	(20)
microscopy	cost effective	requires skilled personnel	
GeneXpert MTB/Rif	high sensitivity	very expensive, not cost-effective	(19)
enzyme-linked immunosorbent assay (ELISA)	cost effective and fast methodology	complex assay handling, require trained staff	(21)
ECL	high sensitivity with broad dynamic range, simple and fast analysis methodology	complex equipment required for point-of-care applications is impractical or expensive	(22, 23)

## Synergetic Relationship between Biosensors and Electrochemiluminescence

**Figure 1:**
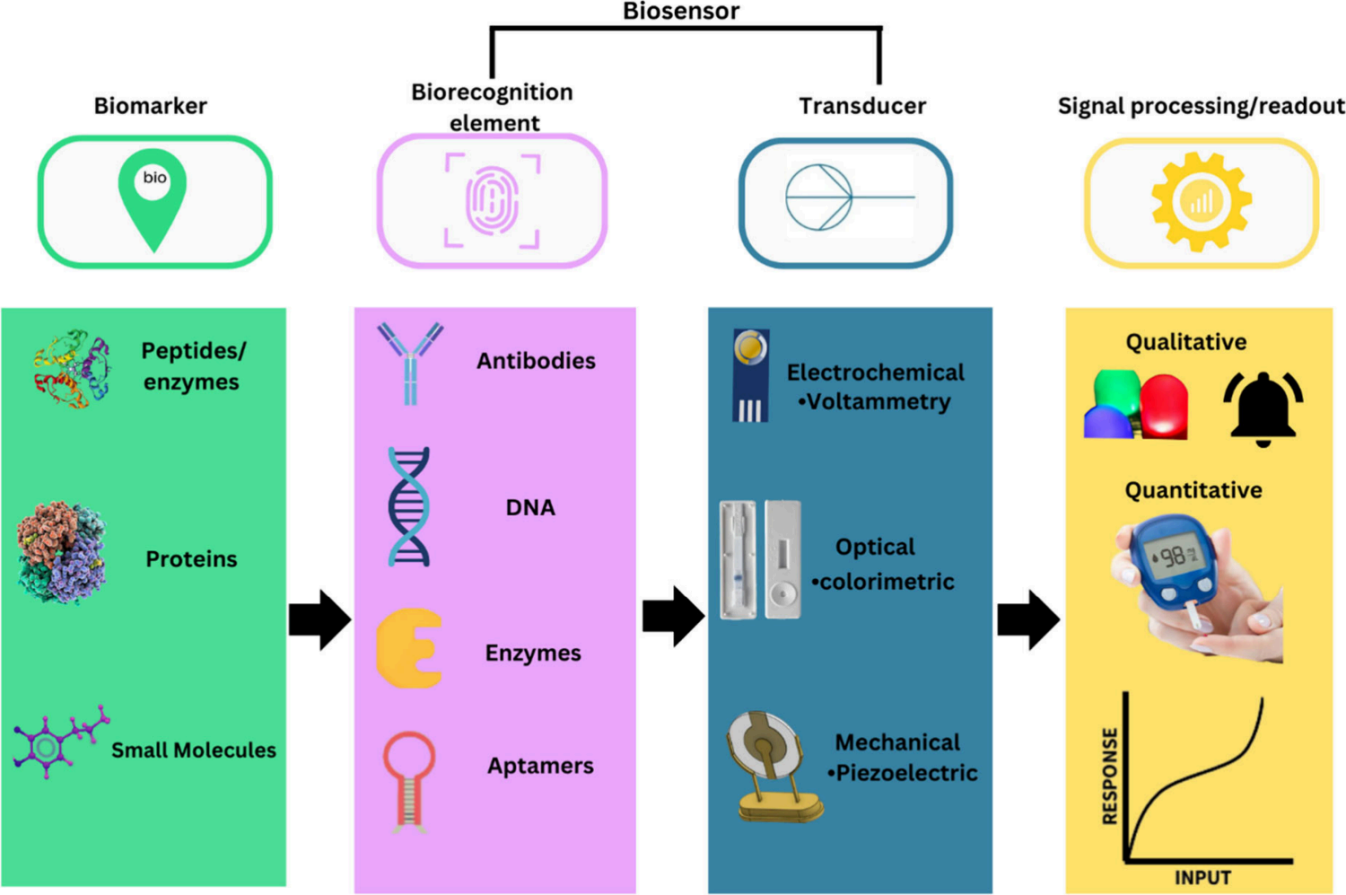
Typical elements and working principles of a biosensor.

A biosensor is a self-contained device that has the ability to provide quantitative analytical information through biospecific interactions between a biomarker and a biorecognition element, which is in direct contact with the transducer, as depicted in Figure 1.^24^ Combining ECL as a transducer for biosensors allows one to leverage the unique-light emitting properties of the technique. Thus, the biosensor detection scheme becomes highly responsive to specific measurements that enables the detection and quantification of biomarkers with precision. ECL biosensors also have access to advantages, namely, low cost, wide linear dynamic ranges, ease of use, capable of being miniaturized, and high device sensitivity attributes.^25,26^

**Figure 2:**
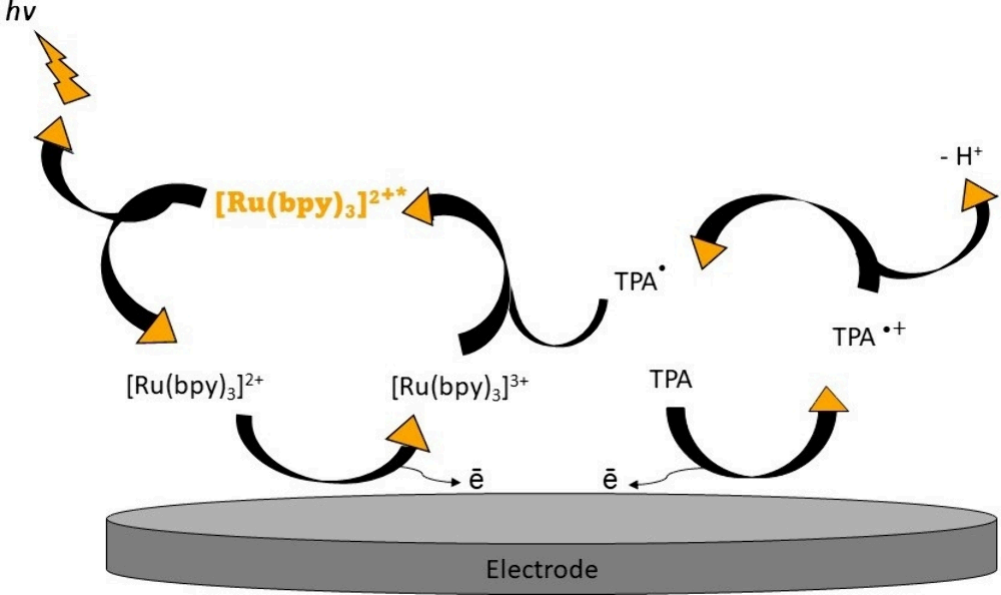
Graphical illustration of ECL showing the tris(2,2′-bipyridine)ruthenium(II) (Ru(bpy)_3_^2+^)/tripropylamine (TPA) coreactant pathway near the surface of an electrode.

ECL utilizes certain light-emitting materials by electrogenerating intermediates that undergo redox reactions to produce the unique luminescence.^27^ The light-emitting materials are called luminophores and is paired with a coreactant species that assist them to produce light.^28^ This is called the coreactant pathway, but there also exists an annihilation pathway from where ECL originated. Moreover, the coreactant pathway provides a stronger and more stable emission that enables a narrow range for the luminescence process.^29^ In contrast, the annihilation pathway requires nonaqueous solvents due to the minimal light emission observed in aqueous media. Additionally, it does not accommodate the conditions for biochemical interactions with biomolecules that typically occur in aqueous solutions.^30^ Therefore, the discovery of the coreactant pathway presented by Bard et al.^31^ paved the way to the applicability of ECL in the research area of biosensors. This breakthrough enabled the signal pairs needed for detection responses to emit light in aqueous solutions. The work led to the exponential increase of publications whereby different biosensors were proposed for detecting various other disease biomarkers,^32−34^ including TB. Further discoveries emerged from the luminophore/coreactant signal pair, consisting of the conventional tris(2,2′-bipyridine)ruthenium(II) (Ru(bpy)_3_^2+^) and oxalate material, i.e., Ru(bpy)_3_^2+^/oxalate presented by Bard. For instance, Leland et al.^35^ explored tripropylamine (TPA) as the coreactant, yielding the Ru(bpy)_3_^2+^/TPA signal pair to produce ECL. Another popular conventional signal pair is the hydrogen peroxide (H_2_O_2_) coreactant in the presence of luminol or, otherwise, the luminol/H_2_O_2_ pair.^36^ These systems are commonly used in ECL biosensors for TB biomarkers due to their simple mechanism. However, it is important to note that newer types of luminophores and coreactants as signal pairs have been explored, though their use in the TB biomarker domain remains limited. To highlight emerging alternative signal emitters, we encourage the reader to explore additional literature.^37−41^ The coreactant mechanism operates on the principle that luminophores undergo either an oxidation or reduction, simultaneously with the coreactant, at the electrode surface. The coreactant produces intermediates that allow it to act as a strong reductant or oxidant for the luminophore. This depends on if the luminophore is more electrophilic, it will pair well with a coreactant capable of producing a strong reductant, or vice versa. Thereafter, the coreactant intermediates engage in a redox reaction with the luminophore intermediates, resulting in its excitation.^42^ As an illustration, the famously used Ru(bpy)_3_^2+^/TPA signal pair mechanism is shown in Figure 2. Herein, an anodic potential sweep is necessary to start the reaction. Both materials Ru(bpy)_3_^2+^ and TPA are typically introduced in solution and oxidized. The strong reductant intermediate produced by TPA undergo redox transfer reactions with the Ru(bpy)_3_^2+^ luminophore and enters an excited state. The luminophore transitions from its excited state to the ground state and emits light.^43,44^

## Clinical Relevance

The review of current ECL-based biosensors for TB biomarkers necessitates a structured approach. For this reason, the viability is first assessed through literature, i.e., based on studies of whether the proposed marker is useful. A threshold or cutoff value will be valuable for the TB-specific biomarkers when reviewing and providing insight into the biosensor’s specifications in later discussions.^45^ This threshold can guide the clinical relevance assessment of the ECL-based biosensors listed in the Review targeting the specific TB biomarkers. The purpose of the cutoff values is to be viewed as a point of reference that was taken from various agreeable cited sources. Moreover, given the dual nature of certain biomarkers utilized for diseases other than TB, the cutoff value enforces a TB-focused approach. Furthermore, this section also aims to identify the market-available tests that are designed to target established TB biomarkers distinguished from the ones that are still in the laboratory testing phase. For example, later in the manuscript, additional TB biomarkers detected using the ECL technique will be identified. These biomarkers, though diagnostic, are considered emerging, as they are not yet detected by commercial TB tests.

## Tuberculosis Biomarker Viability Inspection

C-reactive protein (CRP), as the name implies, is a protein naturally present in the human body.^47^ Healthy levels typically fall within the agreed range of 0–10 mg/L. However, this range can vary considerably and is largely influenced by lifestyle factors or unrelated chronic conditions. These include obesity, diabetes, lack of exercise, smoking, or unhealthy habits in general. In most healthy individuals, the CRP levels tend to be below 3 mg/L.^47^ Notably, the CRP levels can elevate significantly in response to infections, inflammation, or tissue injuries.^48^ This underscores CRP’s utility as a diagnostic biomarker, particularly in TB-specific cases.^49^ Calderwood et al.^50^ has indicated reasonable sensitivities and specificities for CRP as a biomarker. In the study involving 932 individuals, 255 had TB and 389 had TB coexisting with HIV. The CRP levels proved informative and correlated to the infected persons with TB. Others like Soedarsono et al.^51^ assessed the CRP levels in TB patients undergoing treatment. Their findings revealed a decline in CRP levels as patients received treatment. Interestingly, some patients’ levels eventually returned to normal in those who completed their treatments. Furthermore, other studies have demonstrated the usefulness of CRP elevation in pulmonary TB cases when coupled with symptom screening.^52,53^ While the aforementioned provides insights into the role of CRP in TB testing, what would be valuable is the establishment of a threshold value. This manuscript suggests a cutoff value for CRP at 10 mg/L, a recommendation supported by various cited sources.^54−58^ Since CRP is not exclusive to TB, other diseases such as pancreatic disorders or Covid-19 have different thresholds such as 190 mg/L or 40 mg/L, respectively.^59,60^ There are cases where diseases might share a common threshold, like TB’s 10 mg/L. Although, where diseases share the same CRP threshold, it is important to conduct symptom screening tests to differentiate between them.^61^ These tests help determine if the symptoms are more indicative of TB or another disease. Despite this, the 10 mg/L threshold for TB-specific cases is valuable. It serves as a benchmark for reviewing a biosensor’s linear range or limit of detection (LOD) in later discussions. Herewith, thresholds would be the primary focus.

When a host detects the presence of TB bacteria, certain immune cells, such as T helper cells, release the interferon-γ (IFN-γ) cytokine as part of the hosts defense mechanism.^62^ This happens because IFN-γ is required to activate other immune cells, such as macrophages, to coordinate their efforts to combat the infection.^63^ Consequently, the production of IFN-γ in response to the TB bacteria makes it a valuable diagnostic marker. The elevated levels of the IFN-γ biomarker are associated with the TB disease, in contrast to healthy controls, which are less than 13 pg/mL.^64^ The commercial methods, such as the enzyme-linked immunosorbent assay (ELISA), are generally used for the cutoff establishment of biomarkers, as observed in the supporting literature mentioned thus far. Notably, the cutoff value of 14 pg/mL or greater is recommended and approved by the Food and Drug Administration (FDA).^65,66^ However, a study reported a cutoff value of 28 pg/mL that had advantages over the recommended 14 pg/mL for the IFN-γ biomarker.^67^ Further research is needed to support this increase in the cutoff value. By using the standard cutoff value of 14 pg/mL, it minimizes ambiguity and improves the clinical significance in detecting TB. For instance, in cases of extrapulmonary TB, research has shown IFN-γ levels in the pleura to be around 60 pg/mL.^68^ This highlights how various types of TB can be differentiated based on IFN-γ levels. The primary focus remains on pulmonary levels due to their higher occurrence, hence, the suggested level.

The engulfment of TB mycobacteria by the macrophages secretes tumor necrosis factor alpha (TNF-α) among other cytokines. This aids in other complex activation of other immune cells or cytokine mechanisms. This assists in effective phagocytosis to try and destroy the invading bacteria.^69^ The normal values of the TNF-α cytokine are reported to be 1.3 pg/mL in serum.^70^ The cutoff values using the commercially available testing did not show a consensus value unlike before. It would be best to decide based on the notion of who had better sensitivity and specificity percentages. This is generally reported based on the receiver operating curve analysis (ROC) of from which the cutoff values are chosen.^71^ The reason these are reported is because the WHO has a defined criteria for the sensitivity and specificity of diagnostic tests. These percentiles are 90% sensitivity and 70% specificity for detecting the TB disease.^72^ The cutoff value reported for TB-specific TNF-α within the manuscript is the suggested 5 pg/mL, with corresponding sensitivities and specificities of 85% and 70%.^73^ A noteworthy observation is that the cutoff value may be selected based on the goals and context of the study one wishes to undertake since the percentile combination directs these.^74^

The interaction of the transmitted bacilli of TB and its complex interaction with the macrophages causes one of the cytokines interleukin-6 (IL-6) to be released. In turn, these cytokines signal other cells to eventually destroy the bacteria. Stimulating the macrophages starts an inflammatory response and releases IL-6, which can be used as a diagnostic biomarker for TB.^75^ Although no established cutoff value for IL-6 seems to be found, the working range of experiments is found to be within the pg/mL levels, i.e., 4.7–300 pg/mL for TB.^76^ Others reported 4.3 pg/mL (linear range 0.5–24 pg/mL) with healthy controls of 0.05 pg/mL.^77^ Likewise, another reported 22.3 pg/mL cutoff value presented a sensitivity of 77.78% and a specificity of 74.36%.^78^ The suggested cutoff value for TB is the recommended 22.3 pg/mL. This is based on the good sensitivity and specificity percentiles achieved.^79^

There are also deoxyribonucleic acid (DNA) biomarkers, such as the insertion sequence 6110 (IS6110), that are used to detect the TB bacteria, within a host that is unique to members of the mycobacterium complex (MTB).^80^ Unlike before, there is no need for a cutoff value because the IS6110 presence is a direct indicator that someone has the infection.^81^ Instead of a cutoff value, the LOD which is the lowest amount of analyte that can be detected by the instrument, could be beneficial in the clinical relevance when comparing the ECL-based biosensors in later discussions.^82^ This will enable the comparison of the lowest amounts of IS6110 DNA that can be detected by the biosensors. For e.g. the use of a commercial polymerase chain reaction (PCR) instrument had LOD of 0.03 pg/mL whereby this range could serve as a valuable reference.^83^ In subsequent discussions, better LODs using the ECL technique will be observed. Different to the IS6110 TB biomarker, there are instances when TB becomes resistant to the drugs commonly used for treatment. This includes rifampin or isoniazid, among others, whereby the rifampin cannot successfully target the rpoB gene. This is due to the mutations found within its structure causing the drugs to become resistant.^84^ Therefore, the rpoB gene that is found within mycobacteria TB can be used for multidrug-resistant TB (MDR-TB) diagnostics.^85^ Similar to IS6110 in terms of LODs, the biosensors are evaluated based on this parameter instead of a cutoff value.

## Biomarkers Used for Commercial Diagnostic Tests

Interferon gamma release assays (IGRA) are widely used in healthcare facilities to probe the IFN-γ biomarker to test for TB.^86^ Notably, the IGRA test does not directly measure the TB organism, but indirectly measures the cellular immune responses to the mycobacterial protein biomarkers or antigens, such as early secreted antigenic target-6 (ESAT-6) and 10 kDa culture filtrate protein (CFP-10), *in vitro*.^87^ For instance, the blood collected from patients is mixed with the antigens derived from the MTB complex. The antigens are responsible for stimulating the release of the IFN-γ biomarker from the T-cells. Additionally, the amount of IFN-γ released in the patients’ samples are then measured from positive and negative controls in order to formulate the results.^88^ There are different types of IGRAs that have different *in vitro* testing that substantiate the measuring of IFN-γ as a prominent biomarker used at clinics, hospitals, or commercially. The explanation of the IGRA mechanism is for the QuantiFERON-TB assay (Cellestis/Qiagen, Carnegie, Australia), but there is also the T-SPOT.TB assay (Oxford Immunotec, Oxford, UK), which is also recommended and FDA approved.^89^ This approval indicates that the product has met the FDA’s standards for safety, effectiveness, and quality.

Lipoarabinomannan (LAM) is another biomarker tested or probed commercially. It utilizes antibodies contained within the design of the diagnostic test that has affinity for LAM.^90^ LAM forms a part of the cell wall of the mycobacteria which allows it to be readily detected in urine, allowing for a noninvasive procedure *in vitro*.^91^ Commercially available tests include the ELISA sandwich antibody test by companies such as Chemogen Inc. (South Portland, ME, USA) or Clearview TB ELISA by Alere Inc. (Waltham, MA, USA), all detecting LAM in the urine. Likewise, the Alere Determine TB LAM Ag test is also a lateral flow device created by Alere Inc. that uses the same preparations of the Clearview TB ELISA, but in a point of care device.^92^ Speaking of lateral flow devices, other commercially available tests are the TB Ag MPT 64 rapid tests manufactured by Standard Diagnostics Inc. (Seoul, South Korea). The test uses high affinity antibodies against the *Mycobacterium tuberculosis* protein 64 (MPT 64) antigen as a TB biomarker.^93,94^ Distinguished from antigens, the detection of antibodies as biomarkers using the Standard Diagnostics Inc. rapid test is also used.^95^ For instance, in the serum of TB patients, the amount of antibodies is specific to antigens, i.e., the 38 kDa antigen (a specific protein from the mycobacterium) will be high, where many of these tests are premodified with antigens that are affinitive for the antibody.^96^ Thus, the lateral flow devices measure the immunoglobulin G (IgG) and immunoglobulin M (IgM) as biomarkers in the blood, which are specifically produced in response to the antigen.

There is also the popular GeneXpert MTB/RIF test (Cepheid, Sunnyvale, CA), which is a nuclei acid amplification test on the market. It monitors the RNA polymerase beta subunit gene (rpoB) as a biomarker in raw sputum samples.^97^ Additionally, it monitors TB drug resistance by detecting where mutations occur along the 81 base pair core template of the rpoB gene.^98^ Since the rpoB gene is the biomarker, five DNA molecular beacon probes are designed to overlap the entire gene template, differentiating mutated from nonmutated forms, subsequently identifying patients that are resistant to the drug or not.^99,100^

There are various biomarkers that were not included under the discussion section prior as they are still in their laboratory testing phase. Some of the tests mentioned are endorsed by the WHO, such as IGRA, where the diagnostic test is designed for probing the IFN-γ at the present time.^101,102^ Notably, another WHO report shows 19 other commercially available diagnostic tests for TB. These are offered by different companies and manufacturers that use the IgG antibody or MPT 64 antigen as the TB biomarkers.^103^

## Electrochemiluminescence-Based Biosensors for Tuberculosis Biomarkers

The review on different ECL biosensors emphasizes the importance of the luminophore material in the presence of the coreactant. Most efforts are directed toward the various ways the luminophore is utilized for an ECL signal. For instance, directly labeling or tagging the bioreceptors with luminophores is one method.^104^ Labeling antibodies is often achieved through the use of a sandwich configuration or hybridization when using DNA sequences. Moreover, the usage of quencher materials tailored to the biorecognition elements is often used for a controlled signal detection.^105^ Such methods are called label-based methods while when the bioreceptor is not labeled, the approach is referred to as label-free. The label-based ECL biosensors monitor the signal indirectly since the luminophore is attached to the bioreceptor. In contrast, the label-free ECL biosensors directly monitor the signal resulting from the binding between the bioreceptor and the biomarker, as the luminophore acts as a probe in the solution.^106,107^

**Figure 3:**
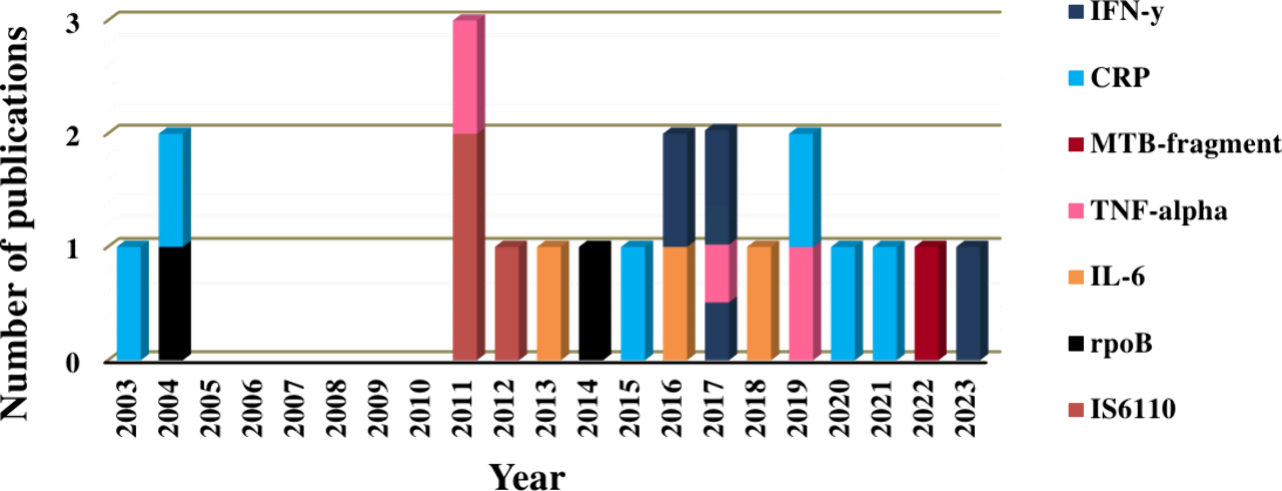
Publications per year for different TB biomarkers used in ECL-based biosensor technology.

In this section, the discussions that will follow for each biomarker will be based on 4 criteria: (1) Inspection on the available ECL-based biosensors for TB published in the literature; (2) Contrasting and emphasizing the different methods followed for an ECL signal; (3) Reviewing the TB thresholds for each ECL-based biosensor listed; (4) Contrasting the ECL-based biosensor with alternative biosensor methods. Notably, for this Review our primary goal is to extensively cover ECL-based TB biomarkers. Thus, contrasting other biosensor transducers to ECL for each biomarker is merely aimed at highlighting the availability of the alternative biosensor method and to illustrate the relative performance in terms of LOD. We include selected articles on alternative biosensors to demonstrate scenarios where these methods do not outperform ECL as well as cases where they do. This is to illustrate the pitfalls observed at times with ECL and how other researchers might propose improvements on the work in cases where alternative biosensors perform better. Moreover, that ECL is not the only technique that can be used, but it is an excellent technique that needs consideration. In this Review, a tally of 21 articles were critically reviewed based on the number of publications per year to identify specific TB biomarkers detected using ECL (refer to Figure 3). A noticeable trend for a specific TB biomarker can be observed from the graph, i.e., 2011 had the most published articles of which one article covered TNF-α and two of the articles published IS6110, respectively. In cases such as in 2017, one publication used two biomarkers showing the simultaneous detection strategy of biomarkers, as denoted by the color scheme. Furthermore, Table 2 summarizes all the subsequent information on the identified TB specific biomarkers using the ECL technique. It is worth mentioning that most articles have not explicitly stated and designed their biosensor for TB. This Review takes that into account and lists them due to the dual nature of certain biomarkers, such as CRP, and based on the evidence in the clinical relevance section that shows their potential use as emerging TB biomarkers.

**Table 2:** ECL-Based Biosensors for the Detection of Various TB Biomarkers^a^

						analytical parameters		
biomarker	substrate	configuration	ECL luminophore - amplification strategy	label-based or label-free	co-reactant	linear dynamic range (LDR) pg/mL	limit of detection (LOD) pg/mL	refs
CRP	Au electrode	sandwich immunosensor	Ru(bpy)_3_^2+^	label-based	TPA	10^6^–10^7^	10^6^	(109)
	Pt electrode	sandwich immunoassay	Ru(bpy)_3_^2+^ -MB@polystyrene bead	label-based	TPA	10^4^–10^7^	10^4^	(111)
	GCE	sandwich immunoassay	Ir-dmpq	label-based	TPA	0–10^3^	3 × 10^3^	(112)
	Au electrode	sandwich immunosensor	Ru(bpy)_3_^2+^ - AuNPs	label-based	TPA	10–10^6^	4.6	(113)
	Pt electrode	sandwich immunosensor	[Ru(bpy)_2_PICH_2_]^2+^	label-based	TPA	10^–3^–10^5^	3 × 10^–4^	(115)
	Nafion (PtNWs/TiNTs) modified Pt electrode	sandwich immunosensor	luminol	label-free	(PtNWs/TiNTs)	5 × 10^4^ to 6 × 10^9^	1 × 10^4^	(118)
IL-6	RGO/Fe_3_O_4_/PDDA/CdSe modified GCE	immunosensor	CdSe	label-free	K_2_S_2_O_8_	2–10^4^	0.65	(121)
	(GO/PANI/CdSe) modified GCE	immunosensor	CdSe	label-free	K_2_S_2_O_8_	0.5–10^4^	0.17	(122)
	Ru(bpy)_3_^2+^@AMCs modified GCE	immunosensor	Ru(bpy)_3_^2+^@AMCs	label-free	H_2_O_2_	10^–5^–10^4^	3.5 × 10^–6^	(123)
IFN-γ	(AuNP@MB/GO-PANI-NF) modified GCE	sandwich immunosensor	CdS - MBs	label-based	K_2_S_2_O_8_	0.1–500	0.03	(125)
	SPE/AuNPs	sandwich immunosensor	Ru(bpy)_3_^2+^	label-based	TPA	2.57–5000	5.7	(126)
TNF-α	PAB electropolymerized modified Au electrode	sandwich immunosensor	CdTe - (Si/PGMA)	label-based	K_2_S_2_O_8_	10^2^–10^6^	7	(128)
	electrodeposited AuNPs modified GCE electrode	aptasensor	Ru(phen)_3_^2+^ - GO	label-based	TPA	5–10^3^	1.5	(129)
IS6110	streptavidin-coated AuNPs ITO electrode	hybridization DNA sensor	luminol - AuNPs	label-based	H_2_O_2_	0.27–26	0.17	(132)
	streptavidin-coated AuNPs ITO electrode	hybridization DNA sensor	luminol - AgNPs	label-based	H_2_O_2_	2 × 10^–3^ to 2 × 10^–2^	8 × 10^–4^	(133)
	streptavidin-coated AuNPs Au electrode	hybridization DNA sensor	ABEI - AuNPs	label-based	H_2_O_2_	2 × 10^–2^ to 25	8 × 10^–3^	(134)
rpoB gene	silicon PIN photodiode patterned with an Au electrode	hybridization DNA sensor	Ru(bpy)_3_^2+^	label-based	TPA	not specified	6.27	(137)
	Ru-GO modified ITO electrode	hybridizationDNA sensor	[*cis*-RuCl_2_(bpy)_2_] - GO	label-free	TPA	7 × 10^2^ to 7 × 10^5^	2 × 10^2^	(138)
MTB DNA fragment	((Ru(dcbpy)_3_^2+^/BPEI/NCNDs)/Nafion/PtNPs) modified GCE	DNA sensor	Ru(dcbpy)_3_^2+^	label-free	(NCNDs-BPEI)	5 × 10^–4^ to 9 × 10^3^	2 × 10^–5^	(140)
IFN-γ and IL-2 (dual-platform electrode)	electrodeposited (RGO/AuNPs) modified ITO electrode	sandwich immunosensor	CQDs - MB@Au and luminol - MB@Au	label-based	K_2_S_2_O_8_ and H_2_O_2_	0.01–1000	10^–2^	(143)
IFN-γ, TNF-α, and IL-2 (triple-platform electrode)	bare ITO	sandwich immunosensor	CQDs - MB@Au, CdS - MB@Au, and luminol - MB@Au	label-based	K_2_S_2_O_8_, K_2_S_2_O_8_ and H_2_O_2_	1.6–200	1.6	(145)

a Indium tin oxide (ITO); AuNPs – gold nanoparticles (AuNPs); deoxyribonucleic acid (DNA); silver nanoparticles (AgNPs); gold (Au); interleukin-6 (IL-6); *N*-(aminobutyl-*N*-ethylisoluminol) (ABEI); graphene oxide (GO); polyaniline (PANI); cadmium selenide (CdSe); reduced graphene oxide (RGO); poly diallyl dimethylammonium chloride (PDDA); iron oxide (Fe_3_O_4_); glassy carbon electrode (GCE); anatase mesocages (AMC); carboxyl terminated ionic liquid (CTIL); poly(*o*-ABA) (PAB); cadmium telluride (CdTe); silica (Si); epoxy polymerization reaction of glycidyl (PGMA); 1,10-phenoanthroline ruthenium(II) (Ru(phen)_3_^2+^); tripropylamine (TPA); nanofiber (NF); magnetic bead (MB); tris(4,4′-dicarboxylic acid-2,2′-bipyridine) ruthenium(II) dichloride (Ru(dcbpy)_3_^2+^); nitrogen-doped carbon nanodots (NDCND); platinum nanoparticles (PtNP); *cis*-dichlorobis(bipyridine) ruthenium(II) (*cis*-RuCl_2_(bipyridine) ruthenium(II)); positive-intrinsic-negative diode (PIN); polyethylenimine (BPEI); ruthenium(II) tris(bipyridine) (Ru(bpy)_3_^2+^); iridium(III)-2-(3,5-dimethylphenyl quinoline) (Ir-dmpg); platinum electrode (Pt); tris(2,2′-bipyridine) ruthenium(II) (2-(4carboxyphenyl) imidazo [4.5-*f*][1,10] phenanthroline ([Ru(bpy)_2_PICH_2_]^2+^); titania nanotubes (TiNTs); platinum nanowires (PtNWs); carbon quantum dots (CQDS); cadmium sulfide (CdS); interferon gamma (IFN-γ); tumor necrosis factor alpha (TNF-α); screen print electrode (SPE); hydrogen peroxide (H_2_O_2_); potassium persulfate (K_2_S_2_O_8_); interleukin-2 (IL-2); insertion sequence 6110 (IS6110); RNA polymerase beta subunit (rpoB); C-reactive protein (CRP). The original values for the analytical parameters LDR and LOD were converted to the pg/mL for an easier comparison. The units not found in this list was originally reported in pg/mL. Keep in mind the original unit = converted unit. Therefore, 1–24 μg/mL = 10^6^–10^7^ pg/mL; 10^–6^ g/mL = 10^6^ pg/mL; 0.010–10 μg/mL = 10^4^–10^7^ pg/mL; 0.010 μg/mL = 10^4^; 0–3 ng/mL = 0–3 × 10^3^ pg/mL; 1 ng/mL = 10^3^ pg/mL; 5 fg/mL – 600 ng/mL = 10^–3^ – 10^5^ pg/mL; 0.3 fg/mL = 3 × 10^–4^ pg/mL; 0.05 mg/L – 6.25 mg/L = 5 × 10^4^–6 × 10^9^ pg/mL; 0.011 mg/L = 1 × 10^4^; 0.01–1000 ng/mL = 10–10^6^ pg/mL; 0.0005–10 ng/mL = 0.5–10^4^ pg/mL; 0.002–20 ng/mL = 2–10^4^ pg/mL; 10 ag/mL–90 ng/mL = 10^–5^–10^4^ pg/mL; 3.5 ag/mL = 3.5 × 10^–6^ pg/mL; 30 fg/mL = 0.03 pg/mL; 0.1 ng/mL–1 μg/mL = 10^2^–10^6^ pg/mL; 0.005–5 ng/mL = 5–10^3^ pg/mL; 10 fg/mL = 10^–2^ pg/mL. For DNA biomarkers such as IS6110 and rpoB, the LOD in the original unit were converted to pg/mL unit and approximated using the molar mass of the target synthetic DNA strand with 330 g/mol for each nucleotide of the sequence.^108^ For instance, 10^–14^–10^–12^ M = 0.27–26 pg/mL; 6.7 × 10^–15^ M = 0.17 pg/mL; 10^–16^–10^–17^ M = 2 × 10^–3^ – 2.6 × 10^–2^ pg/mL; 3 × 10^–17^ M = 8 × 10^–4^ pg/mL; 10^–15^–10^–12^ M = 2 × 10^–2^ – 25 pg/mL; 3.3 × 10^–16^ M = 8 × 10^–3^ pg/mL; 1 pM = 6.27 pg/mL; 0.1–100 nM = 7 × 10^2^–7 × 10^5^ pg/mL; 0.04 nM = 2 × 10^2^ pg/mL; 50 aM–1 nM = 5 × 10^–4^–9 × 10^3^ pg/mL; 1.4 aM = 2 × 10^–5^ pg/mL.

## Electrochemiluminescence Biosensors for C-Reactive Protein (CRP)

**Figure 4:**
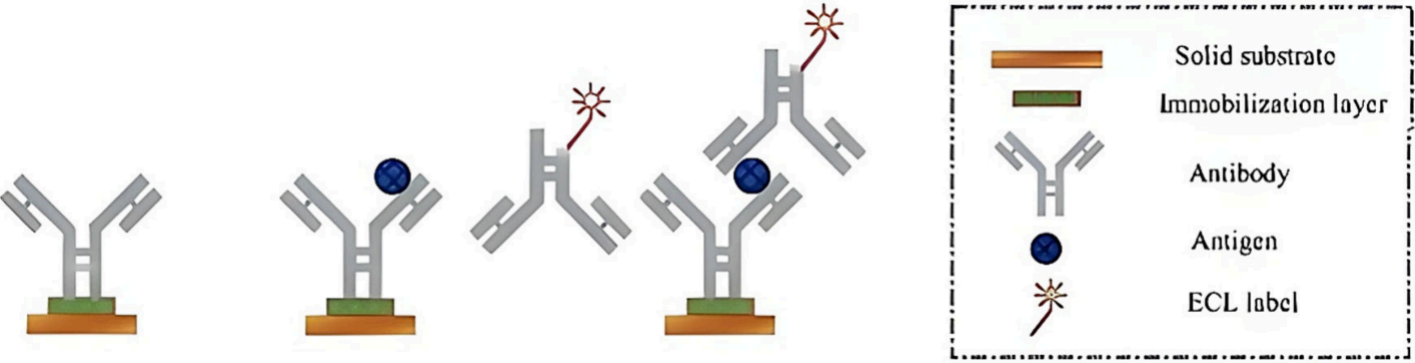
Fabrication of the ECL-based immunosensor for the sensitive detection of CRP. Figure adapted with permission from Bard et al.^109^ Copyright 2003 American Chemical Society.

The majority of the work reported in the CRP section is dominated by the use of label-based/sandwich immunosensor configuration and metal-based electrodes as substrates. For instance, the work produced by Bard et al.^109^ showcased that CRP is detected by their ECL biosensor. The CRP antigen is sandwiched between the capture antibody and labeled Ru(bpy)_3_^2+^ antibody on a gold electrode. In the presence of the TPA in solution, the signal is be produced via the coreactant mechanism. The assembly is depicted in Figure 4. The LOD of this system produced is in the range of 1–24 μg/mL.

**Figure 5:**
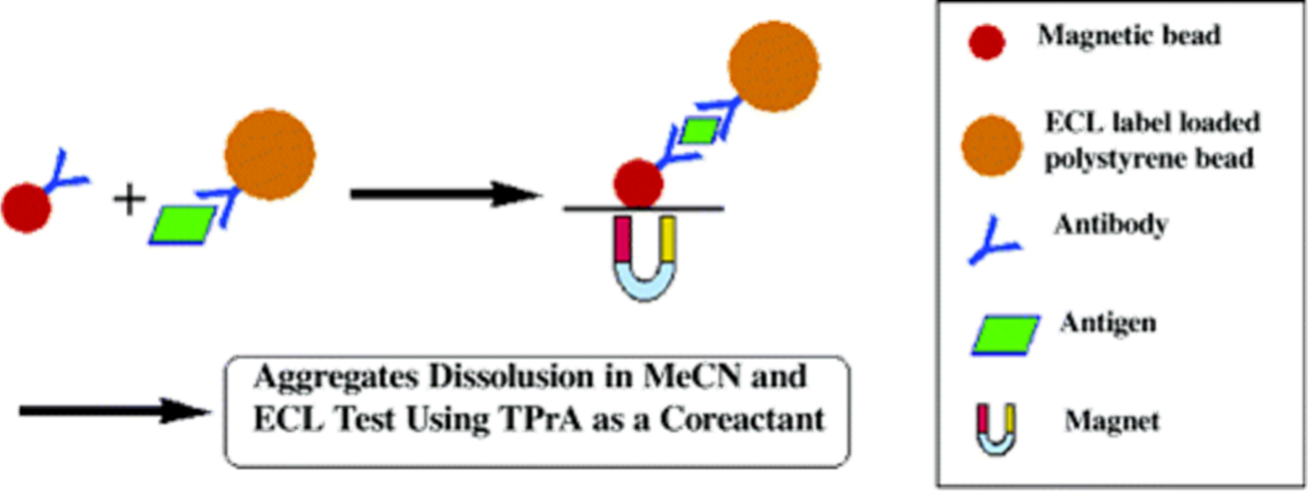
ECL immunoassay using a sandwich configuration using a Ru(bpy)_3_^2+^/TPA system. Reprinted with permission from Bard et al.^111^ Copyright 2004 ACS.

There is also ECL-based assays for detecting the CRP biomarker. In assays the biochemical reactions are first allowed to occur separately and later transferred to the transducer to generate a signal.^110^ Based on the definition provided in the prior introduction, a biosensor biorecognition element should be in direct contact with its transducer element. This statement is made to show that in this Review only biosensors are covered and not all general assays, unless they are used deliberately to illustrate the effect of amplifications strategies or the effect of luminophore material replacements. The work by Bard et al.^111^ detected the CRP biomarker using a sandwich immunoassay by improving the LOD of the Ru(bpy)_3_^2+^/TPA system, as seen from Figure 5. In brief, they included an amplification strategy with magnetic beads containing the Ru(bpy)_3_^2+^ tag. Consequently, more of these Ru(bpy)_3_^2+^ molecules are contained in one bead per antibody immobilized. This facilitated an increase in loading capacity and emission intensity. The ECL generated when these sandwiched entities are transferred to a TPA solution accounted for a 100-fold improvement in the LOD, i.e., 0.010–10 μg/mL.

**Figure 6:**
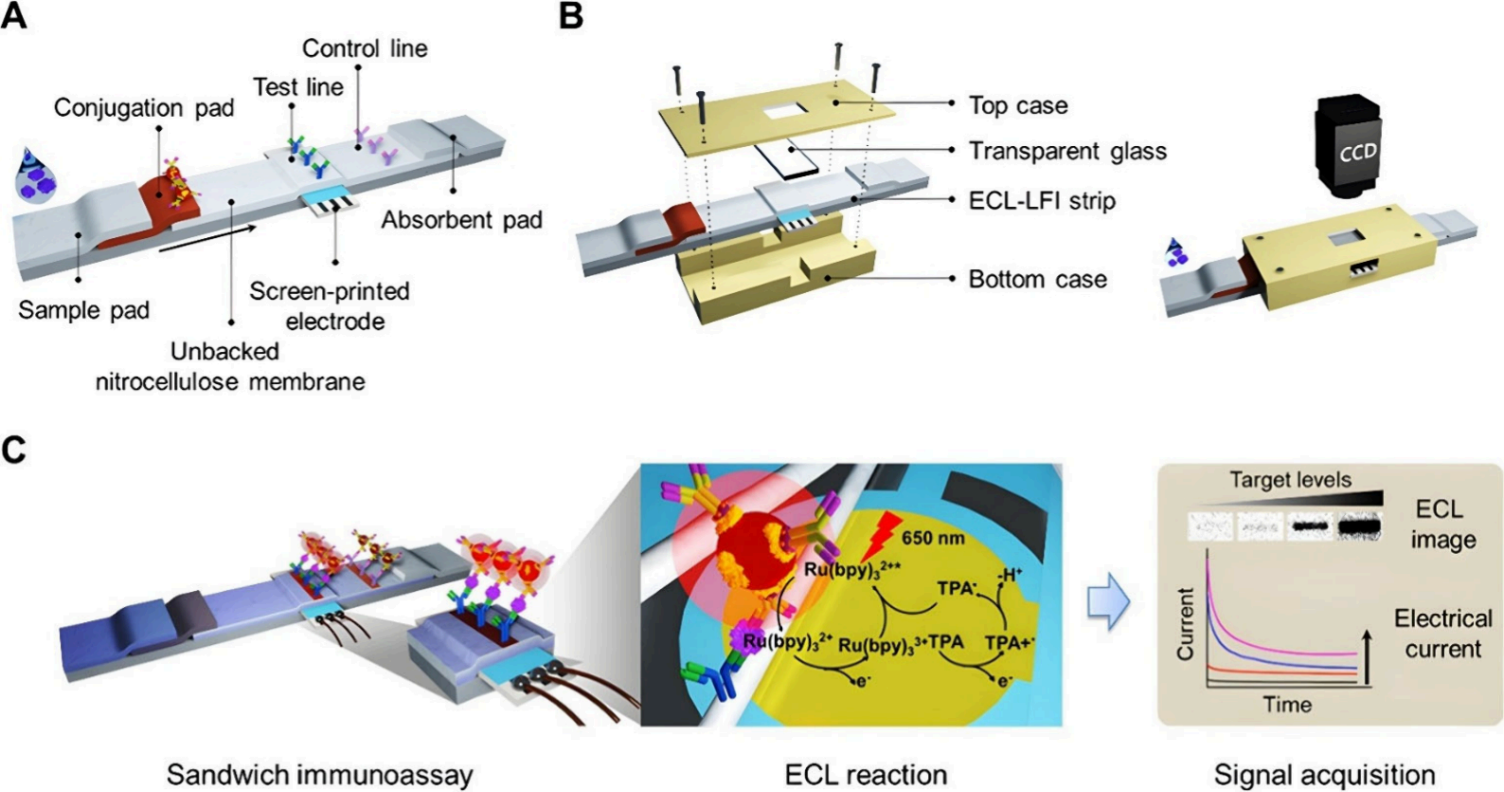
ECL-based immunosensor coupled with lateral flow devices for the detection of CRP. Reprinted with permission from Hong et al.^113^ Copyright 2021 ACS.

Similarly, the work presented by Zhou et al.^112^ made use of the same sandwiched immunoassay. The replacement of the Ru(bpy)_3_^2+^ luminophore to the Ir-dmpq (iridium(III) acetonitrile complex with 2-(3,5- dimethylphenyl)quinoline as the main ligand deviates from the conventional Ru(bpy)_3_^2+^/TPA system. Instead, their use of iridium(III) implies that other metal-based complexes could potentially be explored in conjunction with TPA to detect CRP as a TB biomarker. In their study, the electrochemical reaction between the iridium(III) and TPA produced excited states that emit light that is used for biomarker quantification. The group proposed that iridium produces a higher quantum yield and allows for easier tunable emission properties. By this, they achieved a LOD of 1 ng/mL as opposed Bard et al.^111^ that presented 10 ng/mL (0.01 μg/mL). This shows that incorporating amplification strategies or replacing certain luminophores in the sandwich immunoassays displays a significant contribution in the ECL performance. Similarly, a particular biosensor proposed by Hong et al.^113^ used Ru(bpy)_3_^2+^-labeled gold nanoparticles to enhance the luminophore property. Remarkably, they also illustrated the compatibility of the integration of the ECL technique with a lateral flow device, thus, showcasing the potential use of ECL in hand-held devices for TB, as seen elsewhere.^114^ Essentially, a gold screen printed electrode is situated beneath the test line of a nitrocellulose (NC) membrane. The membrane is also used to immobilize CRP capture antibodies. The conjugation pad consists of Ru(bpy)_3_^2+^-labeled gold nanoparticles attached to the signal capture antibody, forming the probe. The CRP antigen contained within the sample solution will attach to the signal capture probe and flow from the conjugating pad to the test pad. Consequently, forming a sandwich configuration with the probe. Preadded TPA will partake in the electrochemical reaction between the Ru(bpy)_3_^2+^ to produce excited states that emit detectable light for CRP quantification. The novelty of this system as seen in Figure 6 presented a sensitivity of 4.6 pg/mL. Till now, the discussion was driven by the luminophore materials due to the key differences observed in them. The work of O’Reilly et al.^115^ was similar in that it still followed the conventional Ru(bpy)_3_^2+^/TPA signal pair, but they used low molecular weight single chain fragment (scFv) antibodies (typically 25 kDa). By using these scFvs, it alleviates certain challenges experienced with heavy-chain antibodies (∼80 kDa) such as reduced surface coverage, specificity, and orientation problems.^116,117^ The LOD value of 0.3 fg/mL is achieved while still using the electrochemical excitation of Ru(bpy)_3_^2+^ and TPA for the quantification of CRP.

**Figure 7:**
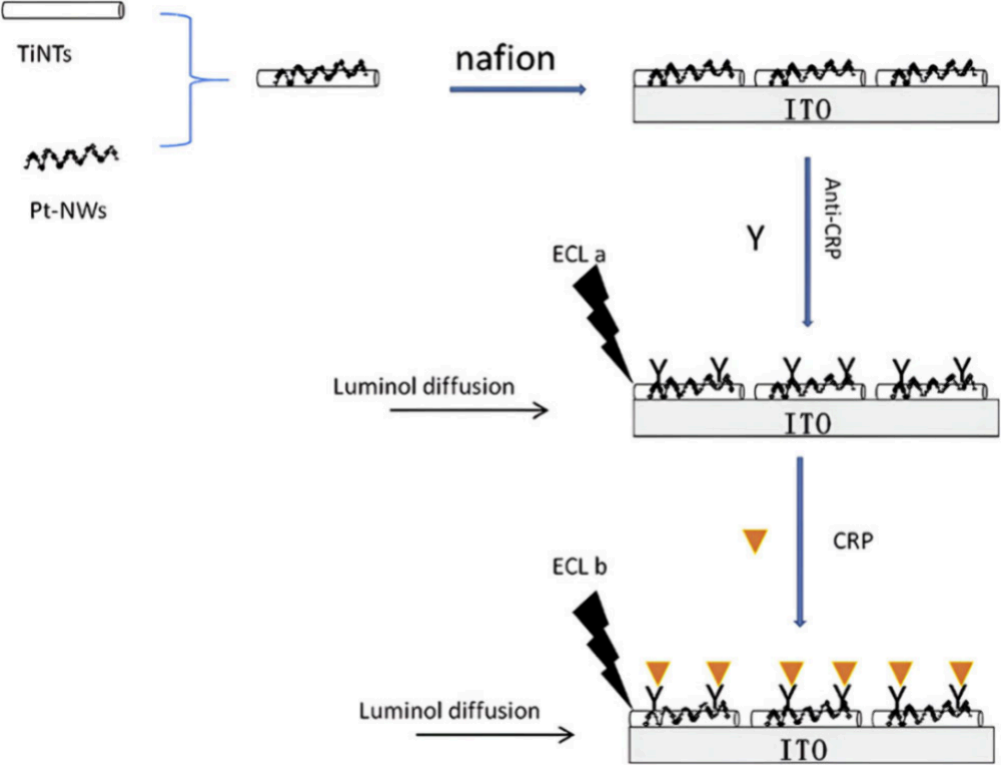
Fabrication of a ECL immunosensor proposed by the authors for CRP detection. Reprinted with permission from Rong et al.^118^ Copyright 2019 Elsevier B.V.

There is also work reported on label-free approaches. The difference is that the luminophore is used as a probe in the presence of the coreactant in solution. The work presented by Rong et al.^118^ utilized such a label-free approach as seen from Figure 7. Interestingly, the common luminol/H_2_O_2_ ECL signal pair is not observed in their method. Rather, a nanocomposite that contained titania nanotubes (TiNTs) and platinum nanowires (PtNWs) was immobilized onto the electrode surface. It acted as the coreactant to oxidize the luminol in solution. This is the first time that a coreactant is immobilized onto a substrate in ECL-based biosensors for TB. The group illustrated that the nanocomposite catalyzes the oxidation of luminol in order to achieve the excited state and deactivation of such to produce the signal. This suggests the possibility of nanomaterials to be used simultaneously as a platform and as a coreactant. However, only if it meets the condition of exciting the luminophore into an excited state. Unlike label-based biosensors that monitor the ECL signal indirectly, the label-free immunosensor configuration monitors the luminophore emission as a probe in solution. By adding the different concentrations of CRP biomarker onto the TiNTs/PtNWs platform, the ECL generated is relayed as a signal in response to the amount of concentration added. The sensitivity achieved from using this strategy was 0.011 mg/L, as reported by the group.

In terms of the clinical relevance of the biosensors mentioned, Bard et al.^109,111^ and Rong et al.^118^ detected CRP within the cutoff value that was pre-established in this manuscript as 10 mg/L for TB. Bard et al.^109,111^ had a linear range of 1–24 and 0.01–10 mg/L, whereas Rong et al.^118^ had a linear range of 0.05–6.25 mg/L, which is acceptable to the 10 mg/L cutoff value. Other sensors produced by O’Reilly et al.^115^ and Hong et al.^113^ are not measuring within the cutoff value for the TB disease. The manuscript reminds the reader of the dual utility of the CRP biomarker in detecting various diseases, e.g., the CRP cutoff value for myocardial infarction has a cutoff value of <3 μg/mL.^119^ In some instances, the sensors outlined within this manuscript were specifically engineered to detect myocardial infarction. Regardless, based on the specifications of the biosensors, their LOD proves satisfactory to detect CRP for TB detection. For instance, Zhou et al.^112^ had 0.01 mg/L LOD with a linear range between 0 and 0.003 mg/L. The sensor is not tested within the TB clinical range but adaptations to this linear range would render it to be useful for TB analysis as an ECL-based biosensor. The same is true for the biosensors covered within this manuscript, which detect CRP. For comparison to alternative biosensor methods, recall the lateral flow devised by Hong et al.^113^ as an ECL-based biosensor. Interestingly, the work of Petruzzi et al.^120^ devised a similar electrochemical-based lateral flow device instead, which followed the same sandwich immunosensor configuration. Since electrochemical methods rely on current, the labeling of the signal capture antibody differs. That is, the enzymatic reaction the label focused on produced a product that is readily oxidized. Regarding the performance metrics of the biosensors, the ECL performed better in the pg/mL range as opposed to the 3 ng/mL of the electrochemical biosensor. Therefore, if the reader is interested in an excellent sensitivity method for a lateral flow biosensor, ECL could be considered. The Table 3 lists all selected alternative biosensor methods that will be referred to in subsequent discussions.

**Table 3:** Different Types of Biosensors for Detecting TB Biomarkers Listed in This Review^a^

biomarker	different type of transducer	configuration	limit of detection (LOD) pg/mL	refs
CRP	electrochemical lateral flow device	sandwich immunosensor	3 × 10^3^	(120)
IL-6	electrochemical impedance	immunosensor	1 × 10^–5^	(124)
IFN-γ	quartz crystal microbalance (QCM)	sandwich immunosensor	5.7	(127)
TNF-α	voltametric	sandwich immunosensor	0.002	(131)
IS6110	fluorescent	hybridization DNA sensor	2.3 × 10^2^	(136)
rpoB	electrochemical	hybridization DNA sensor	3.7 × 10^–3^	(139)

a Quartz crystal microbalance (QCM); deoxyribonucleic acid (DNA); interferon gamma (IFN-γ); tumor necrosis factor alpha (TNF-α); insertion sequence 6110 (IS6110); C-reactive protein (CRP); lateral flow device (LFD). The original values for the analytical parameters LDR and LOD were converted to the pg/mL for an easier comparison. The units not found in this list was originally reported in pg/mL. Keep in mind the original unit = converted united. For DNA biomarkers such as IS6110 and rpoB, the LOD in the original unit were converted to pg/mL unit and approximated using the molar mass of the target synthetic DNA strand with 330 g/mol for each nucleotide of the sequence. Therefore, 3 ng/mL = 3 × 10^3^ pg/mL; 0.01 fg/mL = 1 × 10^–5^ pg/mL; 2 fg/mL = 0.002 pg/mL; 35 pM = 2.3 × 10^2^; 4 fM = 3.7 × 10^–3^.

## Electrochemiluminescence Biosensors for Interleukin-6 (IL-6)

**Figure 8:**
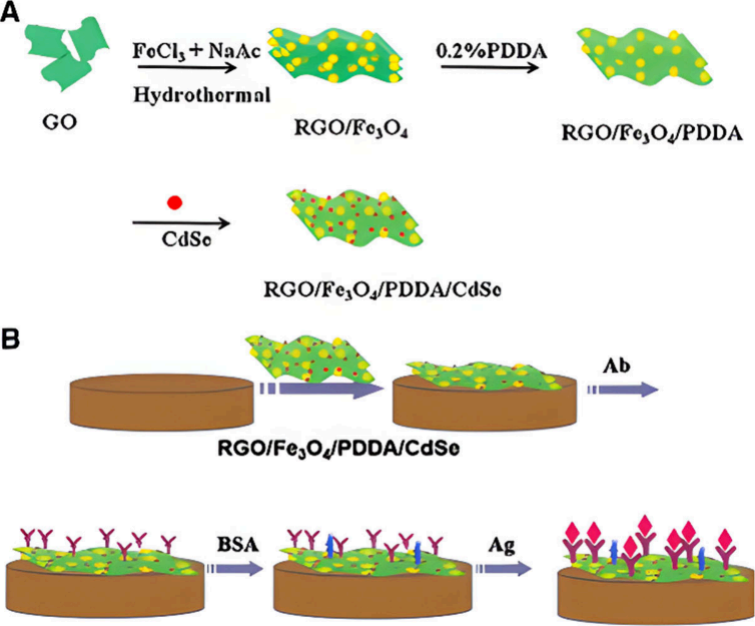
Fabrication assembly of the ECL immunosensor for IL-6 detection. Reprinted with permission from Yang et al.^121^ Copyright 2016 Elsevier Ltd.

**Figure 9:**
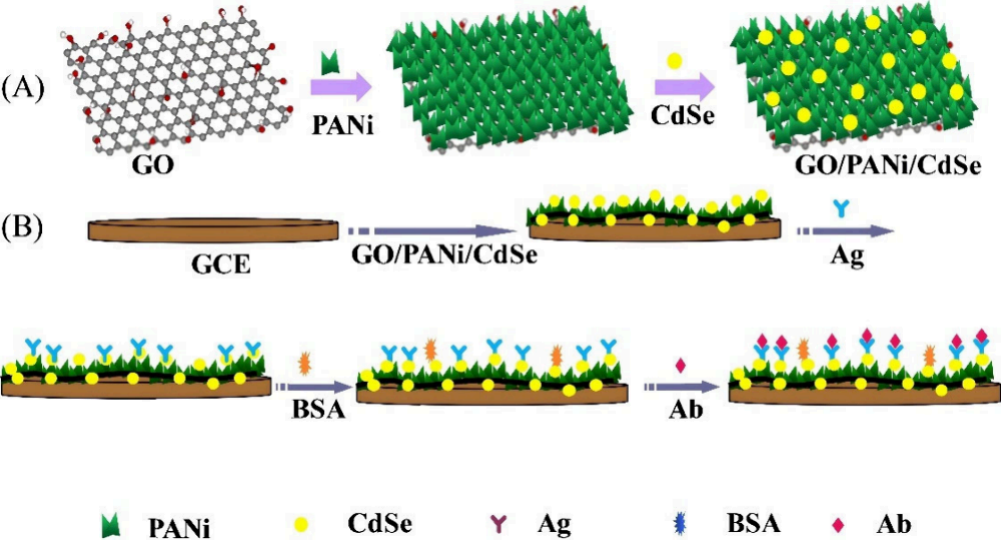
Fabrication assembly of the ECL immunosensor for IL-6 detection. Reprinted with permission from Liu et al.^122^ Copyright 2013 Elsevier Ltd.

The available work on the IL-6 TB biomarker primarily consists of published studies on label-free ECL immunosensors. The authors’ approach focused on the immobilization of luminophore materials onto the substrate surface. For instance, the work performed by Yang et al.^121^ showed a label-free approach, where the cadmium sulfide (CdS) quantum dots (QDs) luminophore species were immobilized on the electrode surface, as seen from Figure 8. To illustrate, the nanoprobe was multifunctional, as it consisted of reduced graphene oxide (RGO), iron oxide (Fe_3_O_4_), poly diallyl dimethylammonium chloride (PDDA), and cadmium selenide (CdSe) QDs that yielded RGO/Fe_3_O_4_/PDDA/CdSe. Moreover, the nanoprobe and antibody were immobilized onto a GCE surface in the presence of potassium persulfate (K_2_S_2_O_8_) as a coreactant for ECL emission. The detection mode relies on the redox reactions between the QDs and K_2_S_2_O_8_ by which the ECL light emission is used to quantify the IL-6 biomarker. The LOD of the system had a value of 0.65 pg/mL. Similarly, the work of Liu et al.^122^ in the same research group replaced the Fe_3_O_4_/PDDA moieties in the previous nanoprobe with polyaniline nanowires (PANi) to yield RGO/PANi/CdSe instead, as depicted in Figure 9. Following the same detection mode, their amplification strategy used increased sensor performance in which the LOD was 0.17 pg/mL. Based on the researchers’ results, it can be derived that QDs can be used both to enhance a substrate’s quality and be used as a luminophore in the presence of a typical strong oxidant coreactant, e.g., K_2_S_2_O_8_.

**Figure 10:**
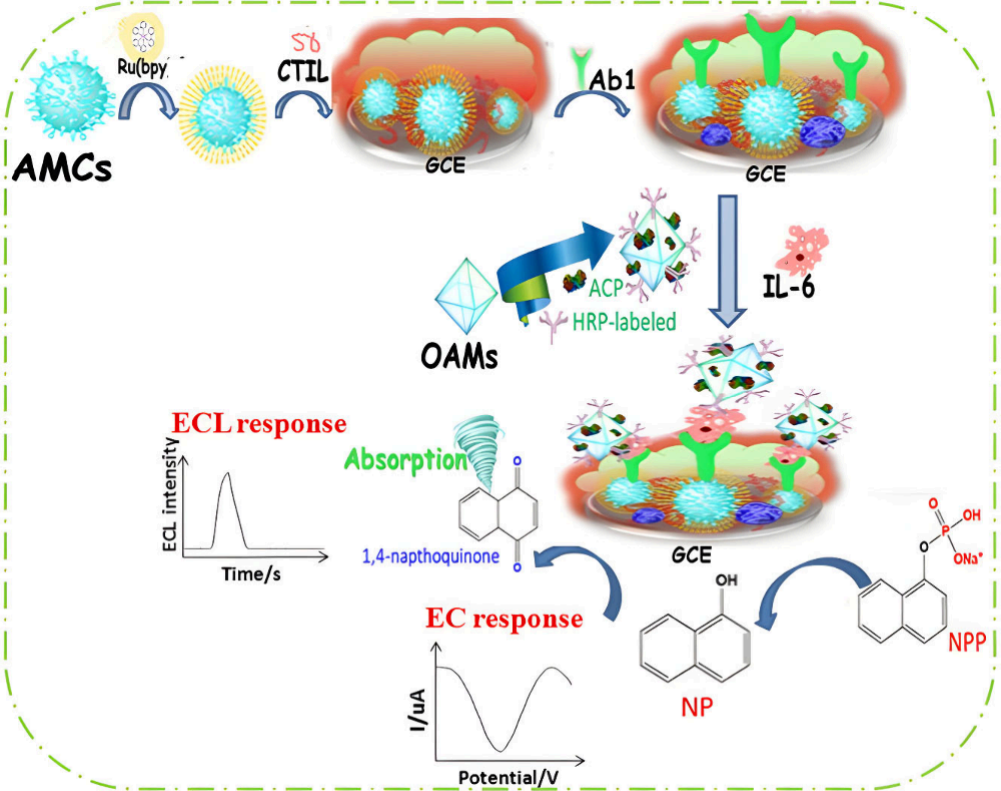
Showing the fabrication and working principle of the dual-responsive ECL-electrochemical biosensor for IL-6 detection. Reprinted with permission from Liu et al.^123^ Copyright 2018 Springer-Verlag GmbH Austria, part of Springer Nature.

The ECL technique is also compatible with the electrochemical technique to produce a dual biosensor. This was displayed by Liu et al.,^123^ as depicted in Figure 10. In brief, a composite material on the electrode surface containing the luminophore Ru(bpy)_3_^2+^@AMCs is used to produce a strong ECL signal, where AMCs is the abbreviation for anatase mesocages. Moreover, a carboxyl-terminated ionic liquid (CTIL) is then added as a linker for the IL-6 capture antibody Ab_1_, yielding Ab_1_/CTIL/Ru(bpy)_3_^2+^@AMCs probe 1. Furthermore, octahedral anatase mesocrystals (OAMs) are used as the matrix for immobilizing acid phosphatase (ACP) and horseradish peroxidase (HRP) with antibody Ab_2_ to form bioconjugates Ab_2_-HRP/ACP/OAMs probe 2. The IL-6 biomarker is sandwiched between the two probes. In the presence of the buffer solution containing 1-naphthyl phosphate (NPP) and H_2_O_2_, 1-napthol is produced via enzymatic hydrolysis of NPP by ACP for the electrochemical signal. Moreover, 1-naphthol can also be oxidized by HRP in the presence of H_2_O_2_ and lead to the amplification of the electrochemical signal. Conversely, the ECL is quenched by the electro-oxidized production of 1-naphthol. Notably, the coreactant for the reaction is not explicitly specified in their work. Thus, it could be either the AMC material, or the H_2_O_2_ (which is a common coreactant in the presence of luminol) that produces the ECL signal. Their results show that when the Ru(bpy)_3_^2+^@AMCs-modified GCE was placed in the buffer solution alone, a strong ECL signal was observed. Interestingly, they managed to immobilize their Ru(bpy)_3_^2+^ in the form of a composite onto the substrate instead of following the typical Ru(bpy)_3_^2+^ in solution or label-based approach. Applying solid state ECL designs is attractive as the diffusion limitations of Ru(bpy)_3_^2+^ in solution can affect ECL performance in biosensors. The reader is encouraged to use Ru(bpy)_3_^2+^ with emerging smart materials for better ECL performance. The LOD for the dual-responsive system consisting of ECL and an electrochemical method are 3.5 ag/mL and 0.32 fg/mL, respectively. Inspecting the TB clinical ranges of the ECL-based sensors from Yang et al.^121^ and Liu et al.^122^ showcases a wide linear range. Their ranges, 2–20000 pg/mL and 0.5–10000 pg/mL, are within 0.5–24 pg/mL when IL-6 has clinical relevance for indicating TB. However, the dual biosensor by Liu et al.^123^ had no relevant range for TB due to its 35–10 ag/mL range, but it could still serve as a diagnostic test, if adjusted, by measuring the IL-6 concentration in the clinical range of TB. With respect to the alternative biosensor method, the work of Yang et al.^124^ devised an electrochemical impedance sensor for the label-free detection of IL-6. Their work was selected and compared to the works of Yang et al.^121^ and Liu et al.^122^ due to the same label-free method they used in their ECL-biosensor. However, the electrochemical method demonstrated 0.01 fg/mL LOD as opposed to the pg/mL range, as seen previously when discussing the ECL-based biosensors. Based on the LODs, the single-walled carbon nanotubes/gold nanoparticles (SWCNTs/AuNPs) created for the electrochemical sensor could potentially have better properties than the RGO/Fe_3_O_4_/PDDA/CdSe or RGO/PANI/CdSe platforms found for the ECL-based sensors. Alternatively, it may only be effective for the electrochemical sensor and not necessarily with the ECL sensor. Interestingly, the QD luminophore for ECL-based sensors was fixed on the substrate surface. It was not used as a probe in solution, as the electrochemical sensing method would employ ferricyanide. It is worth mentioning that there is no study attempted for ECL-based biosensors for TB, where the quantum dot luminophores are actually dispersed in solution.

## Electrochemiluminescence Biosensors for Interferon-γ (IFN-γ)

**Figure 11:**
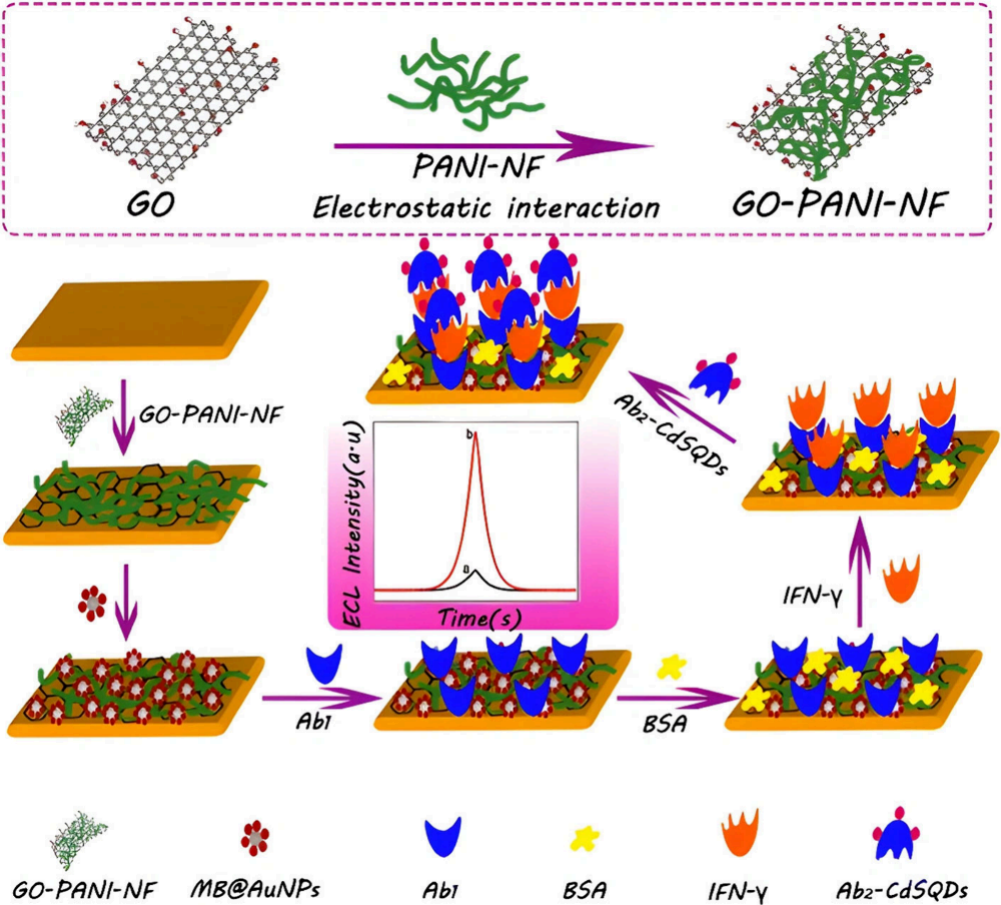
Working principle of the ECL immunosensor for IFN-γ detection. Reprinted eith permission from Zhu et al.^125^ Copyright 2016 Springer-Verlag Wien.

Despite the widespread knowledge of IFN-γ as a biomarker for TB, the work done thus far is very limited and primarily also focused on the sandwich immunosensors. In contrast to the IL-6 section, where the QDs are fixed on the electrode surface, the QD luminophores in this section are attached to the signaling antibody. In one study, Zhu et al.^125^ showcased this as shown in Figure 11. Essentially, gold nanoparticles coated with magnetic beads (AuNP@MB) were attached onto a polyaniline nanofiber (PANI-NF)-doped graphene oxide (GO) GCE surface, yielding Ab_1_/AuNP@MB/GO-PANI-NF. The CdS QDs luminophore attached to the signaling antibody Ab_2_/CdS in the presence of K_2_S_2_O_8_ gave ECL signal response via the typical sandwich reaction. The LOD for the system obtained was 30 fg/mL. In another study, Yuan et al.^126^ focused more on the point-of-care diagnostic ability of the ECL technique, which incorporated a paper-based lateral flow biosensor. The biosensor was comprised of several regions with different pads made with paper that consisted of a sample pad, a conjugation pad, a detection pad, and an electrode region that allowed for ECL emission. The conjugation pad is where the signal antibody is situated and the detection pad is where the capture antibody is placed. Introduction of the IFN-γ is captured and forms a sandwich immunoreaction in the detection pad region, in the direction of the electrode. Upon electrostimulation, the Ru(bpy)_3_^2+^ tagged signal antibody undergoes the ECL reaction in the presence of TPA. The LOD for the sensor is 2.57 pg/mL, having linear range 2.57–5000 pg/mL. This value proved to be acceptable in the TB recommended threshold value of 14 pg/mL. Similarly, the linear range 0.1–500 pg/mL by Zhu et al.^125^ also fell within the cutoff value. Regarding the alternative quartz crystal microbalance (QCM) biosensor method by Pohanka et al.,^127^ the research group labeled the secondary antibody with gold nanoparticles for the signal response instead. More specifically, the sandwich that was formed caused a decrease in the oscillation frequency. Thus, following a similar sandwich configuration detection strategy, they obtained a LOD of 5.7 pg/mL. This is another illustration of how the same detection strategy, while having a different biosensor transducer, yields different LODs.

## Electrochemiluminescence Biosensors for Tumor Necrosis Factor-α (TNF-α)

**Figure 12:**
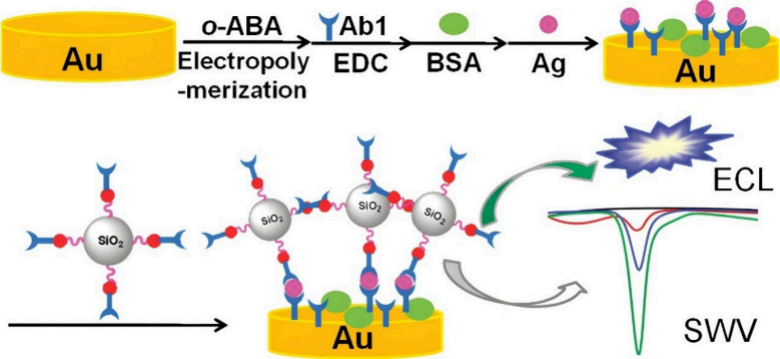
Working principle of the ECL immunosensor for TNF-α detection. Reprinted with permission from Yuan et al.^128^ Copyright 2011 ACS.

The main biosensor configuration observed for detecting the TNF-α TB biomarker was attaching the luminophore to the biorecognition element, i.e., label-based approach. The significance of the work done by Yuan et al.^128^ is the introduction of using a cadmium telluride (CdTe) QD as a luminophore material. The nanoprobe consists of using silica nanospheres as a support for the employment of an epoxy polymerization reaction of glycidyl methacrylate (PGMA). This was to capture the CdTe QD luminophore and to conjugate the nanoprobe to the secondary antibody (Ab_2_) to yield Si/PGMA/CdTe/Ab_2_ for signal detection. Moreover, the capture antibody is immobilized onto a gold electrode by electropolymerizing it with poly(o-ABA) (PAB) film. In Figure 12, the TNF-α antigen sandwich configuration occurs while the CdTe QDs in the nanoprobe and K_2_S_2_O_8_ as the coreactant react, enabling the quantification of TNF-α based on the ECL emission produced. The LOD of the system reported is 7 pg/mL.

**Figure 13:**
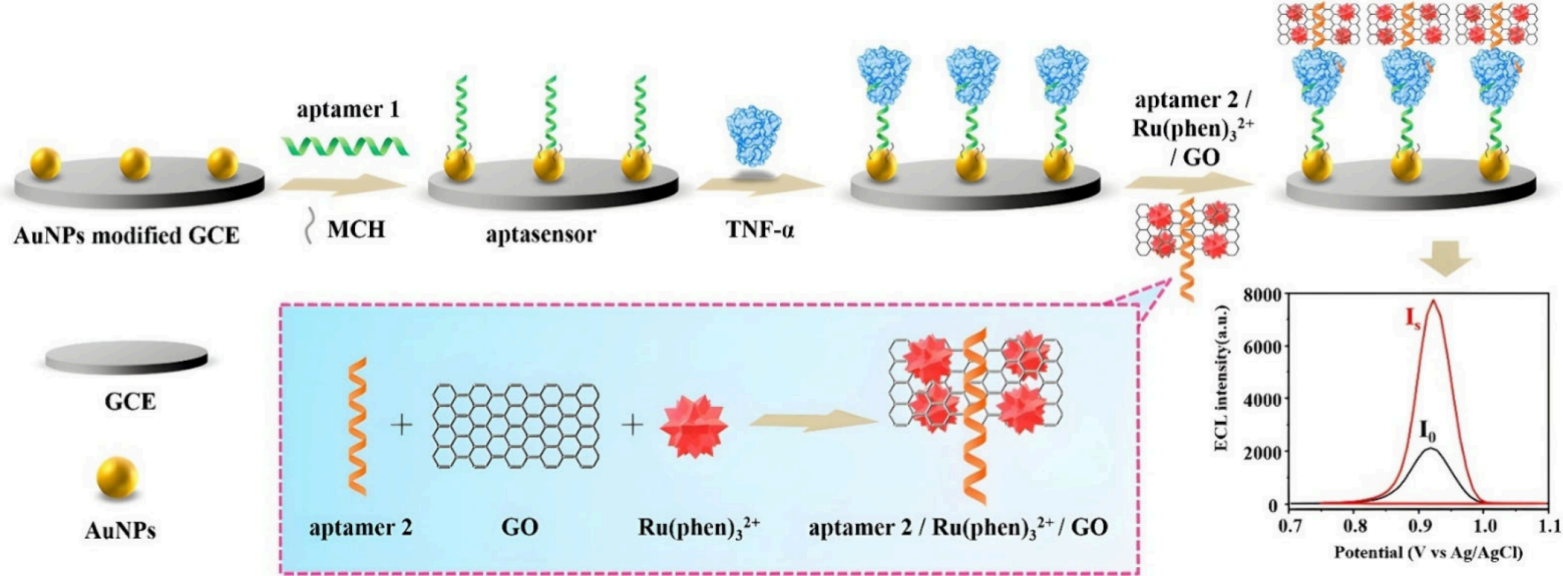
Fabrication and working principle of the ECL aptasensor for TNF-α detection. Reproduced with permission from Gao et al.^129^ Copyright 2019 ACS.

The unconventional work done by Gao et al.^129^ is appealing in that they used two aptamers to sandwich the TNF-α antigen instead of antibodies, as depicted in Figure 13. In aforementioned works contained within the manuscript, this was frequently done using antibodies and not aptamers as biorecognition elements. Nonetheless, the study focused on utilizing two TNF-α specific aptamers, namely, a capture aptamer and signal aptamers for a sandwich configuration. The capture aptamer is immobilized onto a GCE-modified gold nanoparticle surface. The signal aptamer is modified with a nanocomposite luminophore material consisting of 1,10-phenanthroline ruthenium(II) (Ru(phen)_3_^2+^)-graphene oxide (Ru-GO). The detection of TNF-α was based on the redox reaction between the Ru(phen)_3_^2+^ and TPA in solution. The LOD for the system achieved was 1.5 pg/mL. With regards to the TB clinical range, Yuan et al.^128^ measured 100–10^6^ pg/mL levels, which could potentially monitor and differentiate active TB or latent TB using these higher linear ranges.^130^ Furthermore, the biosensor proposed by Gao et al.^129^ had a broad relevant clinical range of 5–5000 pg/mL that includes the suggested 5 pg/mL cutoff value for TNF-α. In contrast to the pg/mL LODs observed with ECL, the study by Yola et al.^131^ demonstrated a 1 fg/mL LOD using the alternative electrochemical biosensor. In brief, they first modified their GCE with gold nanoparticles and multiwalled carbon nanotubes. Thereafter, their signaling antibody was modified with a bimetallic copper/zinc metal organic framework (Ni/Cu-MOFs) for sensor amplification. Consequently, a sandwich type immunoreaction between the capture target antibody and the antigen takes place, whereby the current is produced through H_2_O_2_ as a redox probe. Notably, the Ni/Cu-MOFs antibody enhanced the redox reaction with H_2_O_2_. Similarly, one could extrapolate this idea to explore such novel materials as MOFs for luminophore-labeled antibodies. This could enhance the ECL reaction in the presence of the coreactant, consequently, yielding a better sensitivity biosensor, as illustrated by the electrochemical biosensor.

## Electrochemiluminescence Biosensors for Insertion Sequence 6110 (IS6110)

**Figure 14:**
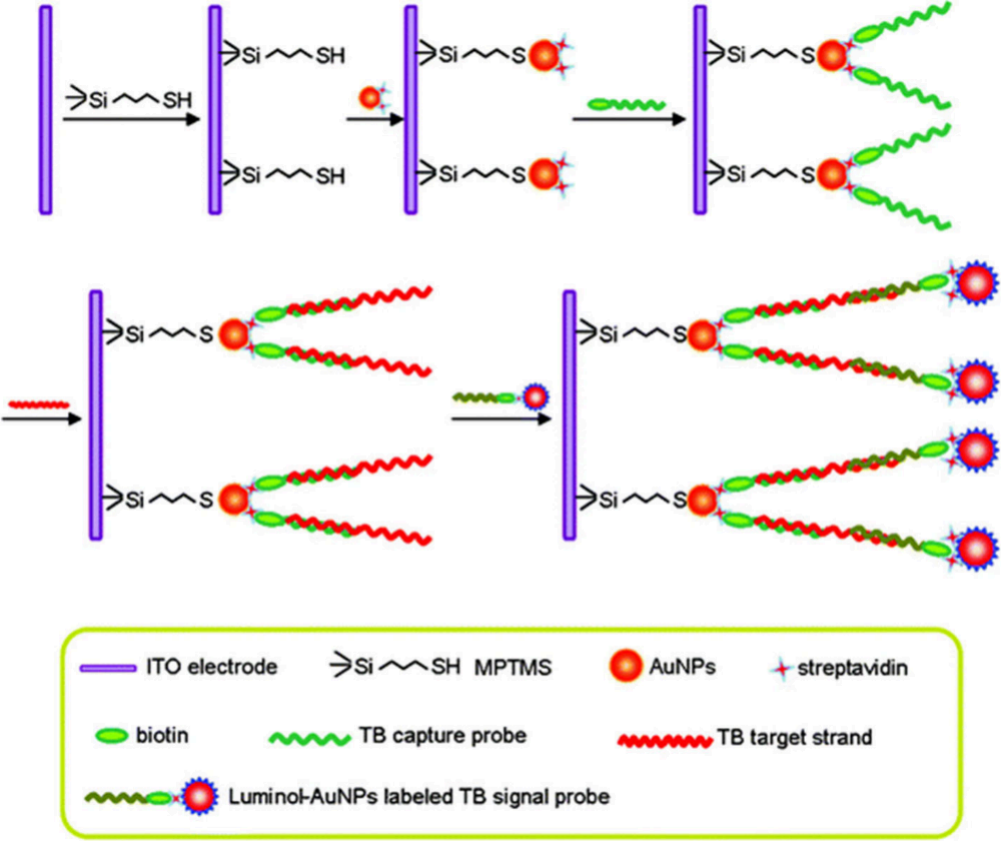
Fabrication of an ECL DNA sensor for IS6110 detection. Reproduced with permission from Jiang et al.^132^ Copyright 2011 RSC.

A hybridization configuration is often followed for the IS6110 biomarker through the use of complementary capture and signal DNA sequences. Notably, the available work for ECL-based biosensors exclusively employed the luminol/H_2_O_2_ pair. Moreover, enhancements to the luminophores performance were achieved by integrating nanomaterials. To demonstrate, Jiang et al.^132^ showcased their novel ECL DNA sensor that utilized a target IS6110 strand as a TB biomarker, as illustrated in Figure 14. The sensor used a complementary capture strand immobilized on an indium tin oxide (ITO) electrode modified with gold nanoparticles. Furthermore, a signal strand was also prepared. The signal strand label consisted of a nanocomposite luminophore material containing luminol and gold nanoparticles. The target strand hybridized with both capture and signal strands, which is complementary to each end of the target strand forming a hybridization configuration. In the presence of H_2_O_2_ as a coreactant, luminol in the signal strand undergoes a redox reaction, producing light emission. This emitted light is then used to quantify the IS6110 target strand.

**Figure 15:**
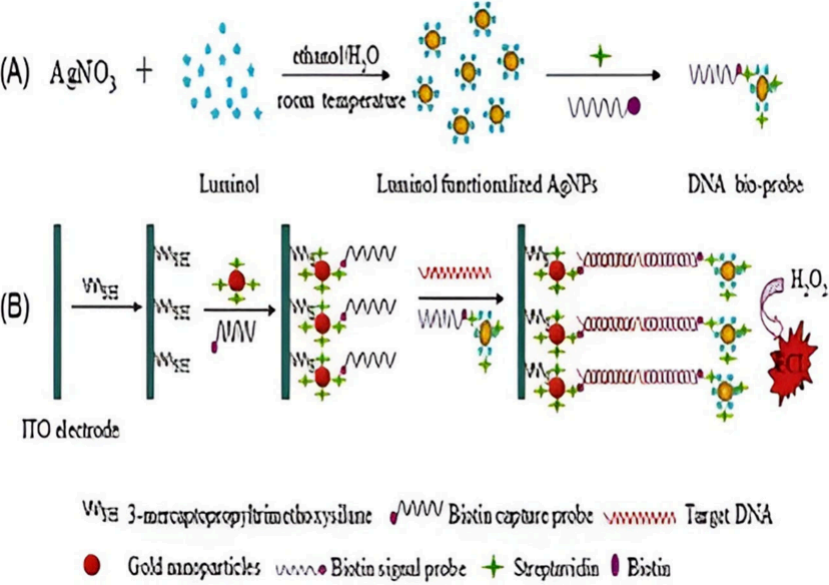
Preparation and fabrication assembly of the IS6110 DNA sensor. Reprinted with permission from He et al.^133^ Copyright 2011 RSC.

**Figure 16:**
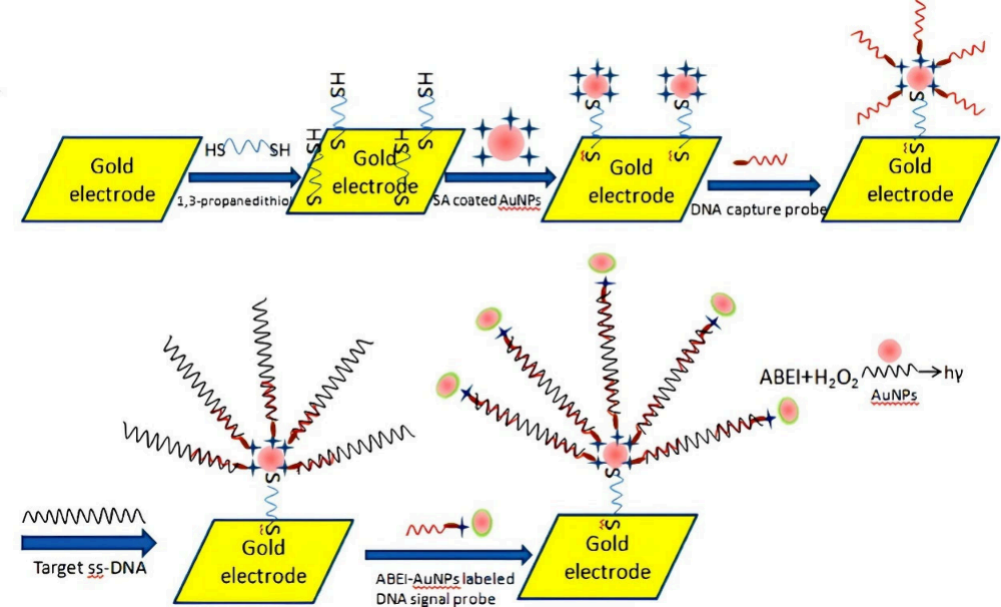
Fabrication process of the IS6110 DNA sensor. Reprinted with permission from Yu et al.^134^ Copyright 2012 Elsevier B.V.

Similarly, the work of He et al.^133^ consisted of a similar set up as that just discussed and followed the same detection mode. However, they used silver nanoparticles with the luminol instead of gold nanoparticles as a signal amplification strategy, as depicted in Figure 15. The differences in their work yielded different analytical performances of both sensors in which the LOD for Jiang et al.^132^ was 6.7 × 10^–15^ M and He et al.^133^ was 3 × 10^–17^ M. It can be deduced that since the signal label was a major difference between the two sensor components, it played a crucial part in terms of sensitivity of the sensors. Furthermore, the work of Yu et al.^134^ also demonstrated interesting results when the nanocomposite relating to the signal strand label was changed. A luminol luminophore derivative was used, namely, *N*-(aminobutyl)-*N*-(ethylisoluminol) (ABEI) functionalized with gold nanoparticles (AuNPs), yielding the nanocomposite ABEI-AuNPs, refer to Figure 16. As a note, the biosensor set up and detection mode was the exact same as Jiang et al.^132^ of the same research group. However, the substrate had changed from ITO to a gold electrode. Through the usage of the ABEI gold nanoparticles as a label for the signal strand, the LOD of 3.3 × 10^–16^ M showed improvement. The usage of the luminol-gold nanoparticulate system did not yield a better result when using luminol-silver nanoparticles as the luminophore material. Inspection based on the LODs in the same unit are 6.7 pM by Jiang et al.,^132^ 0.03 pM by He et al.,^133^ and 3.3 pM by Yu et al.^134^ From these one can observe that the method by He et al.^133^ has the ability to detect the lowest concentrations of analyte. As before, detecting trace amounts of the IS6110 biomarker is crucial because any presence of it in samples is indicative of TB, as documented in the clinical relevance section.^135^ In contrast, the 35 pM LOD achieved through the alternative fluorescent biosensor developed by Liang et al.^136^ was lower. For context, their detection strategy relied on using a metal organic framework and CdTe QDs to provide a quenching effect in the absence of the MTB gene. In the presence of the target, a fluorescence signal is obtained via hybridization of the target to the capture probe. Thus, the ECL biosensor exhibited the lowest LOD that showcases high sensitivity.

## Electrochemiluminescence Biosensors for the RNA Polymerase β Subunit (rpoB)

**Figure 17:**
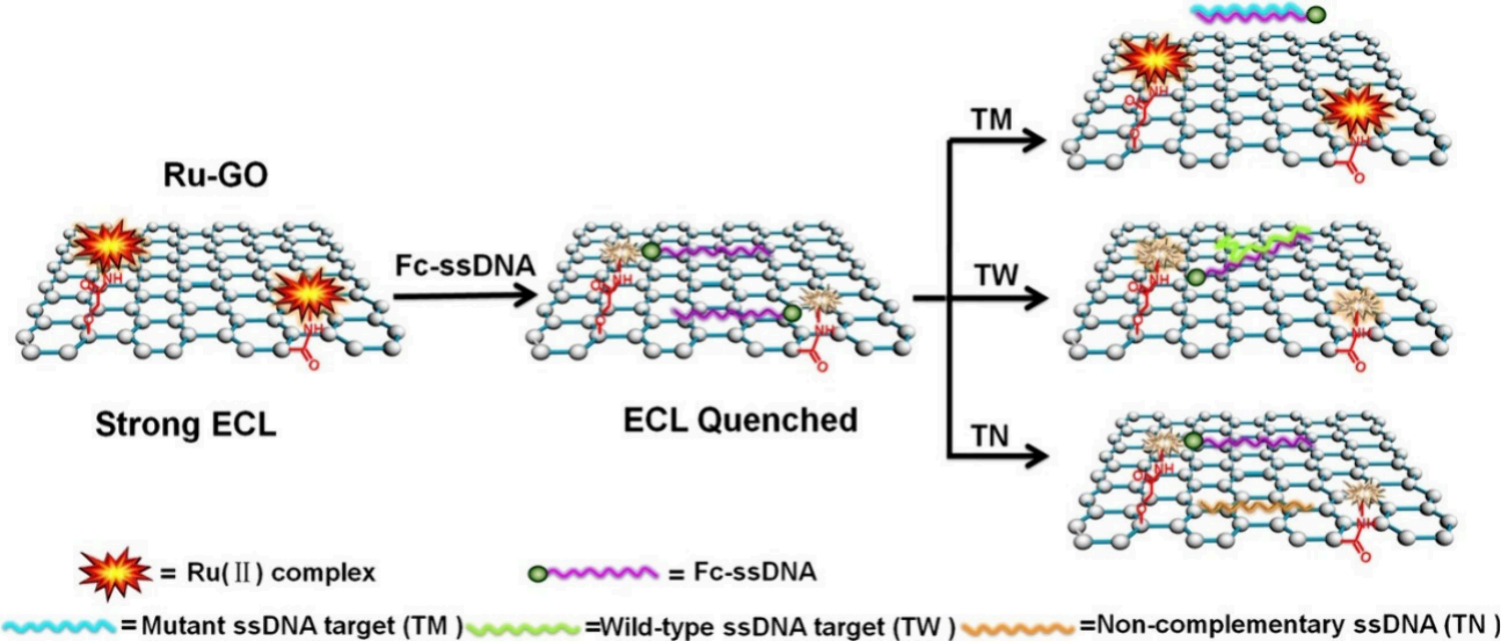
Fabrication assembly of the ECL DNA sensor for rpoB gene detection. Reprinted with permission from Li et al.^138^ Copyright 2014 ACS.

The reports for rpoB focused on the hybridization configuration of Ru(bpy)_3_^2+^-tagged DNA signal strands with capture and biomarker strands. In a study conducted by Sweeney et al.,^137^ the biorecognition element was a single-stranded DNA element that was immobilized on a silicon positive-intrinsic-negative diode (PIN) photodiode (patterned with gold electrodes through lithography methods) via thiol linkage. A target DNA strand is also labeled with Ru(bpy)_3_^2+^ that upon DNA hybridization with a rpoB biomarker strand will produce the required ECL emission in the presence of the TPA coreactant upon electrostimulation. Thus, the detection mode is based on the ECL emission and is related to the amount of rpoB. The LOD for the system produced was 1 pM. In the work of Li et al.,^138^ the sensor construction was based on using Ru(bpy)_3_^2+^ functionalized with graphene oxide (Ru-GO). This acted as a sensing platform wherein ferrocene was attached to the single-stranded DNA capture probe. The ferrocene acted as an ECL intensity quencher to the Ru-GO luminophore species. The ECL interfacial emitter Ru-GO and ferrocene-labeled DNA were immobilized onto an ITO electrode, as depicted in Figure 17. Interestingly, the luminophore is attached to the substrate surface while the TPA coreactant remains in solution, a technique previously observed. This novel approach, which deviates from traditional DNA tagging methods, is applied. Thus, the solid-state Ru-GO luminophore undergoes redox reactions with the TPA to produce the light emission used to quantify the rpoB biomarker. Moreover, the analytical performance obtained, testing for the biomarker using this system, was 0.04 nM. Based on the LOD values, the 1 pM LOD of Sweeney et al.^137^ is the lowest. In comparison to the alternative electrochemical DNA sensor by Haddaoui et al.,^139^ the LOD was 4 fM. They achieved this through the use of a polypyrrole-coated Fe_3_O_4_ nanoparticle-functionalized poly amidoamine dendrimer platform. This again indicates that good sensitivities could be reached using other techniques. Here, the redox probe naphthoquinone is integrated into the platform in a manner similar to how the QDs probe is incorporated, as seen previously. Interestingly, the comparable LODs suggest the adoption of a similar approach by replacing the redox probe with a luminophore material instead, which may be advantageous.

## Electrochemiluminescence Biosensor for *Mycobacterium tuberculosis* (MTB) Fragments

**Figure 18:**
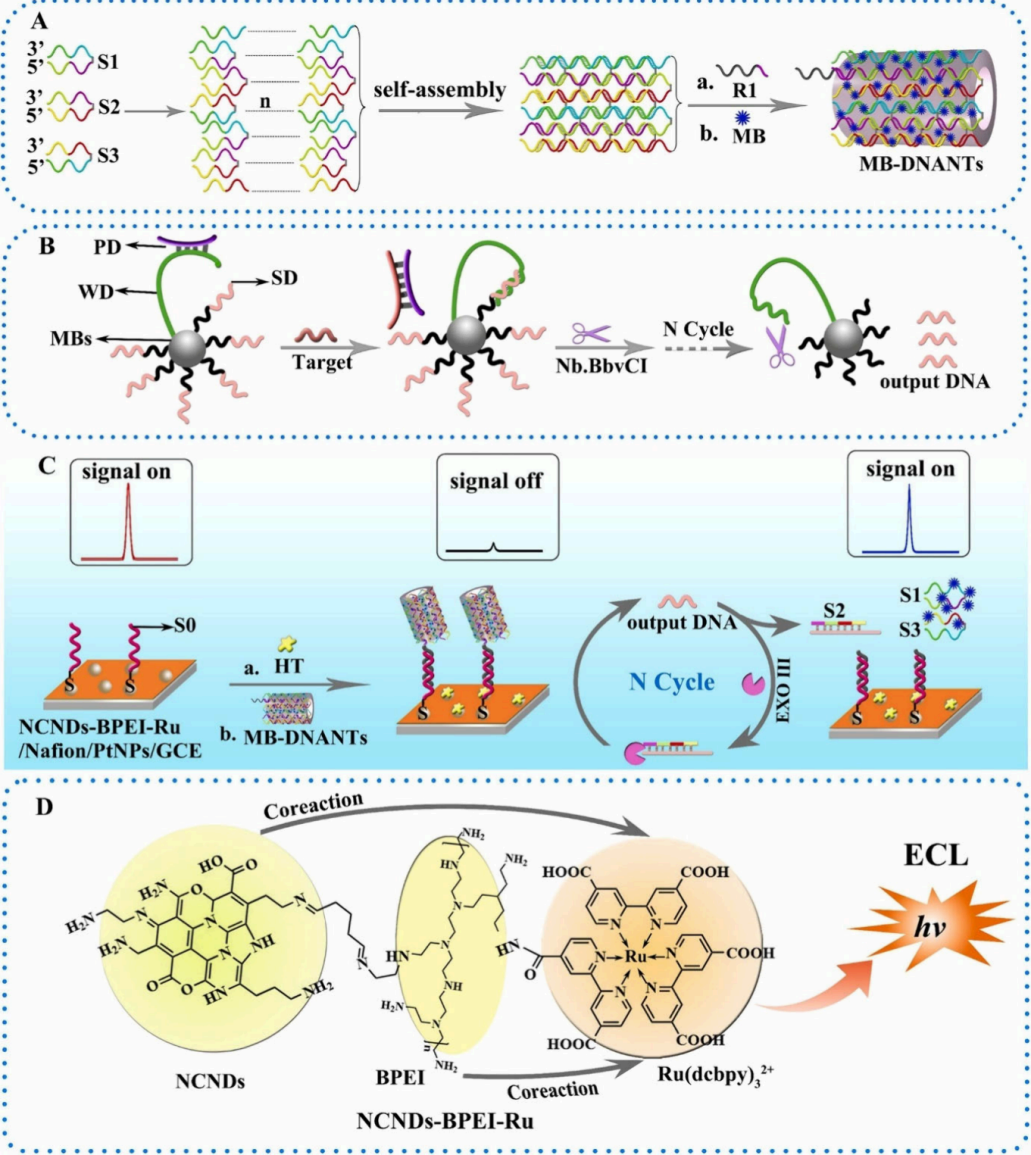
Fabrication and working principle of the ECL self-enhanced sensor using DNA fragment detection for TB. Adapted with permission from Hu et al.^140^ Copyright 2021 Elsevier B.V.

All the documented ECL biosensors for each biomarker have incorporated the luminophore and coreactant as distinct components. However, Hu et al.^140^ made a significant advancement by combining both ECL signal pairs in one material to facilitate the ECL reaction. The sensor consisted of tris(4,4’dicarboxylicacid-2,2’-bypyridyl) ruthenium(II) dichloride (Ru(dcbpy)_3_^2+^) functionalized with polyethylenimine (BPEI) and nitrogen-doped carbon nanodots (NDCNDs), yielding (Ru(dcbpy)_3_^2+^/BPEI/NCNDs). The hybrid material allowed the Ru(dcbpy)_3_^2+^ to act as a luminophore, and the NDCNDs/BPEI moieties completed the role of the coreactant. Nafion is used as a binder to immobilize the material onto the GCE, which has been modified with platinum nanoparticles. The approach outlined was complex, involving two main steps: (1) the DNA nanotubes with methylene blue (MB-DNANTs) were added to quench the ECL signal of NCNDs-BPEI-Ru for the signal response, and (2) the 3D DNA nanomachine (made with DNA-functionalized magnetic beads) can convert trace amounts of target MTB into large amounts of output DNA. Consequently, the MB-DNANTs break apart, which no longer quenches the ECL signal, but restores it because of the hybridization between the output DNA and the DNA in the DNANTs, refer to Figure 18. The overall system showed a LOD in the range of 1.4 aM (attomolar). The LOD produced by the authors was the lowest concentration to be detected and is excellent in terms of the clinical relevance of detecting TB.

## Electrochemiluminescence Biosensors for the Simultaneous Detection of Multiple Tuberculosis Biomarkers

**Figure 19:**
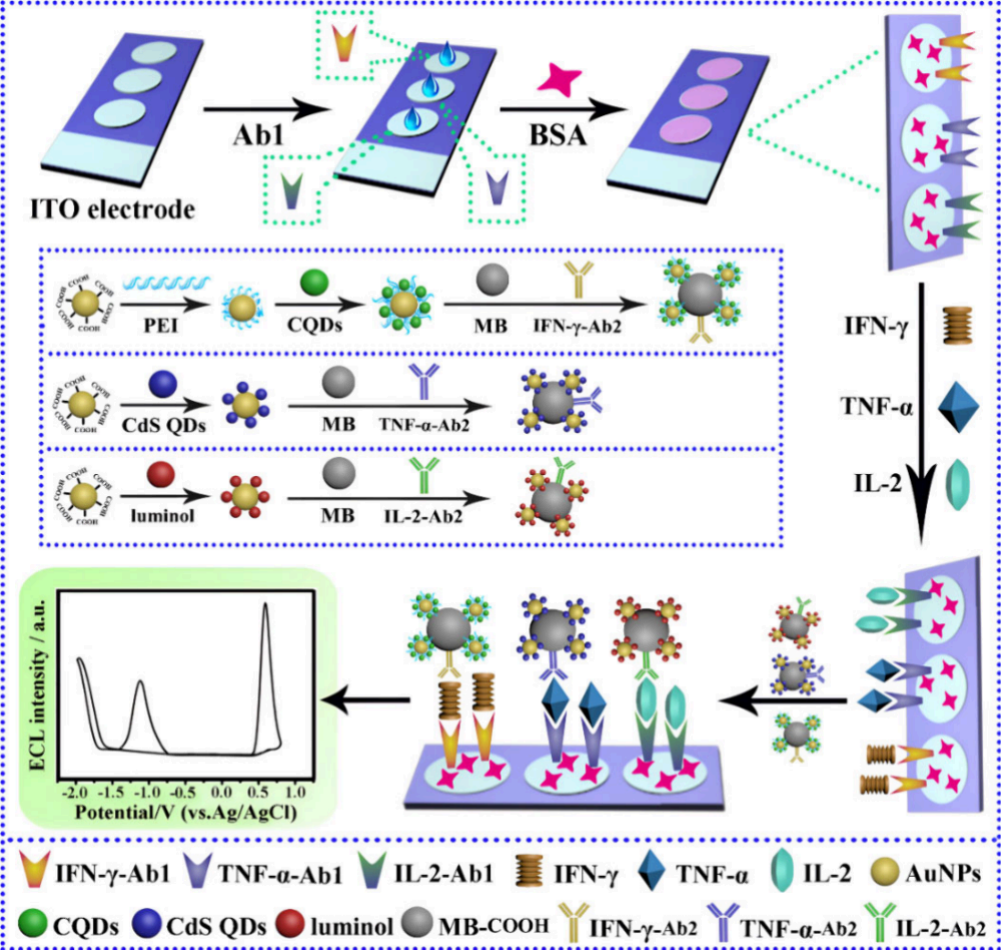
Preparation, fabrication, and working process of the ECL immunosensor multiplexed system for the detection of 3 biomarkers, namely, IFN-γ, IL-2, and TNF-α. Reprinted with permission from Zhou et al.^145^ Copyright 2017 ACS.

Till now, the biomarkers discussed were detected in isolation. The logic behind “simultaneous detection” allows for better accuracy and precision, as other biomarkers are not exclusive to TB. The dual approach would provide more reliable results in confirming the presence of TB disease.^141^ Therefore, ECL also facilitates the use of biosensor platforms for the simultaneous detection of TB biomarkers.^142^ The study presented by Zhou et al.^143^ revealed the possibility of this specifically for TB. The method was based on a dual platform created on a single ITO electrode in parallel, whereby graphene oxide and gold nanoparticles are electrodeposited. This facilitated the attachment of multiple primary antibody capture probes for IFN-γ and interlekin-2 (IL-2) onto adjacent areas of a single electrode. Notably, IL-2 is a diagnostic biomarker for TB. However, it was not covered individually as the others listed in the review due to no work found on ECL-based biosensors for this biomarker. There are studies done to show that elevated levels are found to be related to TB.^144^ Nonetheless, the first probe consisted of magnetic beads functionalized with gold nanoparticles and carbon quantum dots (CQDs), including the secondary IFN-γ antibody attached that yielded MB@Au@CQD-IFN-γ-Ab_2_. Similarly, for the secondary IL-2 antibody, they only replaced the CQDs with luminol, which yielded MB@Au@luminol-IL-2-Ab_2_. In the presence of the respective biomarkers of IFN-γ and IL-2, a sandwich configuration formed in which the luminophores in each probe, such as CQDs and luminol, underwent redox reactions to produce ECL emission with coreactant K_2_S_2_O_8_ and H_2_O_2_, respectively, to quantify the biomarkers. The biosensor system reached a LOD of 10 fg/mL. Interestingly, Zhou et al.^145^ of the same research group extended the biomarker platform to create three adjacent platforms. The achieved LOD was 1.6 pg/mL, see Figure 19. The sensor fabrication was similar to the dual-platform electrode, with the key difference being the addition of an adjacent platform designed to detect the TNF-α biomarker. Moreover, interfacial layers of graphene oxide and gold nanoparticles was not included in the study as before. The capture probes of the primary TNF-α antibody were immobilized in parallel on the ITO electrodes. Furthermore, the signal probe consisted of the functionalization of magnetic beads, gold nanoparticles, and CdS QDs attached to the secondary TNF-α antibody, yielding the nanoprobe MB@Au@CdS-TNF-α-Ab_2_. Notably, the added adjacent platform detection mode relied on the CdS and K_2_S_2_O_8_ to undergo redox reactions for the ECL light emission in order to quantify the TNF-α biomarker. Therefore, it can be deduced that the addition of interfacial layers contributes to the overall sensitivity as opposed to having no interfacial layers for electrode modification. Thus, it could have enhanced the ECL efficiency by the promotion of more electron transfer reactions to take place, of which graphene oxide is a good candidate.^146^ Notably, the same research group, Zhou et al.,^143^ obtained 10 fg/mL with previous methodology. This could be because magnetic beads were on the surface of the electrode, but in the dual platform electrode the magnetic beads were part of the signal probes. This is because of the MB material property that allows for more loading capacity, either for capture probes or signal probes depending on the use, yielding different analytical results.^147^ The linear ranges are clinically relevant in both studies, i.e., in the pg/mL range (0.01–1000 pg/mL for dual and 1.6–200 pg/mL for triplicate platforms) of which the cutoff values of the biomarkers tested by these platforms are within these ranges of 5 pg/mL (TNF-α), 14 pg/mL (IFN-γ), and 10–40 pg/mL for IL-2.^148^

## Reliability Assessment of Electrochemiluminescence Biosensors for Tuberculosis Biomarkers

In the Electrochemiluminescence-Based Biosensors for Tuberculosis Biomarkers section the biosensor setups, configurations, materials, and trends were previously highlighted in the manuscript. However, an evaluation of the reliability of the ECL biosensors for TB biomarkers would provide valuable insights in the future status of TB biomarker detection. This section will highlight a few examples of the ECL biosensors discussed earlier to provide evidence that demonstrates their reliability, refer to Table 4. The discussion will include aspects such as real-sample analysis, stability, selectivity, repeatability, or reproducibility of the ECL biosensors. Different biosensors (based on the ECL biosensors for TB biomarkers covered within the manuscript previously) were analyzed for this discussion and were chosen for their comprehensive approach, addressing all the aspects, such as selectivity, stability, reproducibility, and real-sample analysis, which will offer a more reliable assessment. The reason other ECL biosensors for TB biomarkers in the manuscript were not selected is because they only cover one or two aspects. This will not give an overall reliable assessment because, although a biosensor may show good selectivity, if stability is not included in the study we are limited to showing the true reliability of the biosensor.

**Table 4:** Assessment of Stability/Reproducibility, Selectivity, and Real Sample Analysis in ECL Biosensors for TB Biomarkers^a^

ECL biosensor based on TB biomarker	stability/reproducibility	selectivity/interferences	real sample analysis	refs
CRP biosensor	stored at 4 °C for 10 days, retaining 90% of the original ECL response	albumin, glucose, ascorbic acid, and uric acid were tested at 100× the concentration of CRP	CRP recovery in real serum samples ranged from 93% to 110%.	(118)
IL-6 biosensor	stored at 4 °C for 7 days, retaining original ECL response	glucose, bovine serum albumin, carcinoembryonic antigen, and l-cysteine tested at increasing concentrations	IL-6 average recovery in human serum of 101.23% compared to the ELISA method	(121)
	three measurements had a 4.9% RSD for reproducibility			
IFN-γ biosensor	stored at 4 °C for 10 days, retaining 93.8% of the original ECL response, five measurements had 4.96% RSD for reproducibility	bovine serum albumin, immunoglobulin G, and transferrin. IFN-γ was also mixed with each interferent and tested	IFN-γ recovery in human serum ranged from 96% to 103% compared with the ELISA method	(125)
MTB biosensor	four measurements had RSDs of 2.35% and 1.52%	different interference DNA strands were tested at 100× the concentration of the MTB DNA target strand	MTB recovery in human blood serum ranged from 95.25% to 104.50%,	(140)

a C-reactive protein (CRP); interferon-γ (IFN-γ); relative standard deviation (RSD); interleukin-6 (IL-6); mycobacterium tuberculosis (MTB); enzyme-linked immunosorbent assay (ELISA); electrochemiluminescence (ECL).

**Figure 20:**
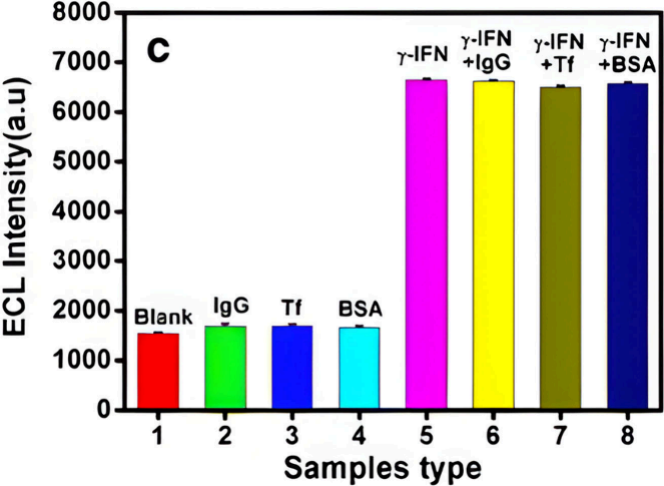
Selectivity of the IFN-γ biosensor evaluation using various samples, including bovine serum albumin (BSA), immunoglobulin G (IgG), and transferrin (Tf), each at a concentration of 100 pg/mL. The test conditions include a blank sample and a mixture containing 100 pg/mL of IFN-γ, along with 100 pg/mL of each interferent: BSA, IgG, and Tf. Adapted with permission from Zhu et al.^125^ Copyright 2016 Springer-Verlag Wien.

A notable observation regarding stability was observed in the ECL biosensors, as seen in Table 4. When subjected to storage temperatures for 7–10 days, the biosensors retained their original ECL response. Specifically, the CRP and IFN-γ biosensors maintained 90% stability. Furthermore, when the biosensors were tested with repeated measurements to assess reproducibility, they exhibited a good relative standard deviation (RSD) value. The RSD reflects the precision of repeated measurements, ensuring consistent and reliable results. Notably, the IL-6 and IFN-γ biosensors achieved an RSD less than 5%. In comparison, the MTB biosensor demonstrated an even better RSD of less than 2%, indicating superior reproducibility. Overall, the ECL biosensors presented are reproducible. To assess the sensor’s selectivity, all biosensors were tested against potential interfering molecules, as shown in the selectivity column of Table 4. The sensors exhibited good selectivity; as in most cases, the concentration of interferences was two to three times higher than that of the biomarkers or the interferences were mixed with the biomarker. For example, Figure 20 shows IFN-γ in the presence of interferences and a mixture of IFN-γ with interferences. The ECL intensity clearly demonstrates the difference, confirming that the sensor is more selective for the IFN-γ biomarker. This was true for the CRP, IL-6, and MTB biosensors. Based on the real sample analysis presented in Table 4, the practicality of the biosensors was further examined in real clinical samples. For instance, the IL-6 biosensor was analyzed in concentrations of IL-6 in human serum. The ECL immunosensor showed satisfactory results, with an average recovery rate of 101.23%, which demonstrates that the IL-6 biosensor could be successfully applied to the clinical detection of IL-6 levels in human serum. Since the recovery rate of a biosensor in real samples indicates how accurately it measures the target analyte, it is compared to a known or spiked amount in the sample. Interestingly, the IL-6 biosensor also had results that are consistent with the first line techniques, such as ELISA, which was also done with the IFN-γ biosensor. This shows good prospects for ECL biosensors for detecting TB biomarkers. Besides human serum, the MTB biosensor was evaluated in human blood serum. The results presented the recovery from 95.25% to 104.50%, suggesting the excellent practicality of the proposed biosensor. This indicates that in different bodily fluids these biomarkers can be detected using the ECL biosensors. Overall, these biosensors illustrate and give us an idea of the reliability and potential of the ECL biosensors.

## Conclusion

One major cause of tuberculosis (TB) mortality is the limitations associated with current detection methods. Over the past decade, electrochemiluminescence (ECL) biosensors have been explored as an alternative detection method for TB biomarkers. In the work covered by this manuscript, 70% of the studies reviewed focused on ECL immunosensors, and the rest focused on DNA biosensors for TB biomarkers, as outlined in the Electrochemiluminescence-Based Biosensors for Tuberculosis Biomarkers section. Within this manuscript, most studies utilized label-based sensors rather than label-free approaches, which are often associated with enhanced sensitivity. This highlights a significant opportunity to explore more label-free approaches. Another key observation from the reviewed studies is the common use of traditional ECL signal emitters, such as Ru(bpy)_3_^2+^/TPA signal couples, for signal generation. In these systems, the biorecognition element is typically tagged with Ru(bpy)_3_^2+^, while TPA is present in solution. Similarly, the luminol/H_2_O_2_ system is frequently employed as a signal emitter. Alternative luminophores, such as quantum dots (QDs), were frequently employed when traditional luminophores like Ru(bpy)_3_^2+^ or luminol were not employed. In these cases, K_2_S_2_O_8_ often acts as the coreactant in solution. Notably, QDs serve a dual purpose, whereby they function as luminophores and provide an enhanced surface area, increased loading capacity, and improved conductivity. Other notable efforts have also been made to deviate from conventional ECL biosensor designs. These innovative approaches include immobilizing coreactant materials on the electrode surface rather than having them present in solution, incorporating luminophore materials with other smart materials to form composites, and adding various novel amplification materials (e.g., nanomaterials) to modify the surface of substrates. Other advancements include the development of self-enhanced luminophore materials and the rare use of a sandwich aptasensor configuration was seen. In terms of biomarkers, traditional biomarkers, such as IFN-γ, LAM, rpoB, IS6110, and MPT64, are commonly used in commercial TB diagnostic tests, as discussed in the Biomarkers Used for Commercial Diagnostic Tests section. However, in ECL biosensor applications for TB biomarkers, only rpoB and IFN-γ have been found (which was limited). This indicates a need for further exploration of additional commercially available biomarkers. Research on CRP, IL-6, and TNF-α as potential TB biomarkers has shown clinical significance. Therefore, these biomarkers should be explicitly investigated for use in TB biosensors. There have also been promising efforts to develop ECL biosensors for point-of-care applications, such as lateral flow devices for CRP and IFN-γ detection, as discussed in the Electrochemiluminescence-Based Biosensors for Tuberculosis Biomarkers section. However, these biosensors are still in the research stage and require further development, particularly for the detection of TB biomarkers. Much more research is needed before such devices can be considered viable for commercial use in TB detection. The challenges include clinical validation, regulatory approval, scalability, and user-friendliness. In all instances, the ECL biosensors demonstrated good sensitivity, successfully detecting the biomarkers. Furthermore, when a reliability assessment was conducted on several of the biosensors discussed in the manuscript under the section Reliability Assessment of Electrochemiluminescence Biosensors for Tuberculosis Biomarkers, the ECL biosensors displayed good stability, selectivity, and the ability to detect real samples in human serum. Therefore, the issue is not whether ECL exhibits these qualities, as its potential has already been advocated for. However, based on all our observations, summarizing points, and identified gaps, the main disadvantages found for ECL biosensors for TB biomarkers are as follows:

(1) Limited exploration of label-free approaches reported. To explore these approaches would be beneficial because they are cost-effective and simpler.
(2) Heavy reliance on traditional ECL signal emitters, such as Ru(bpy)_3_^2+^, which is still considered the gold standard and expensive, limiting innovation in signal generation. Coreactants have been explored minimally, with only one unique case presented in the manuscript having been immobilized onto the electrode surface instead of in solution.
(3) In some instances, smart materials like quantum dots (QDs) are frequently reported as platforms or luminophores. However, they are often expensive and not fully explored in terms of toxicity, especially the cadmium derivatives frequently reported. To explore the use of better smart materials that are environmentally friendly, such as biodegradable polymers, or biomass carbon-derived materials would be better suited.
(4) Limited use of commercially available biomarkers. Only a few biomarkers (rpoB and IFN-γ) are commonly used in ECL biosensors. Focusing on commercially detected biomarkers, which have been validated, would be beneficial. Other biomarkers like CRP, IL-6, and TNF-α have clinical significance, but are underexplored.
(5) Though promising, point of care applications for TB biomarkers are still showing infancy stages of research and require further development, as seen in the lateral flow ECL biosensor device presented. Converting these biosensors into point-of-care devices requires extensive clinical trials and validation and not just proof of concept. Other issues, such as scalability and user-friendliness, also remain issues, particularly in making these biosensors easy to use in the real world. A potential solution could be to integrate phone-based devices with lateral flow devices to detect ECL signals, which could improve usability. Researchers are actively working on this approach, as most ECL device components are not fully portable yet and still very lab-based. In summary, advances in ECL biosensors represent a pivotal shift in TB diagnostics, highlighting a lot of opportunities to investigate additional ECL biosensors for TB, exploring environmentally friendly luminophores and coreactants and commercialized biomarkers.

## Vocabulary

Electrochemiluminescence: This phenomenon consists of the production of light as a result of a redox reaction.
Luminophore: A luminophore is a molecule or functional group in a chemical compound that is responsible for its luminescent properties. In ECL, it emits light upon excitation via an electrochemical reaction.
Co-reactant: A coreactant is a substance that reacts alongside the main reactant such as a luminophore. It often plays a crucial role in facilitating or enhancing the reaction. In electrochemiluminescence, it aids in the generation of the light signal.
Biomarker: A biomarker is a measurable indicator found in bodily fluids such as small proteins called cytokines that show whether a normal, abnormal, or disease process is occurring in the body. It helps in diagnosing conditions and monitoring how well treatments are working.
Biorecognition elements: These are biomolecules such as antibodies, enzymes, or nucleic acids that specifically recognize or detect and bind to target analytes or otherwise biomarkers.
Limit of detection: This refers to the smallest amount of a substance that can be reliably detected by an analytical method. It is a very important parameter in biosensors that evaluates the sensitivity of a detection method or instrument.
Linear dynamic range: This refers to the range of concentrations over which a detection method provides a response that is directly proportional to the concentration of the analyte. Very useful when a known cutoff value is known for a particular analyte that falls within the linear range.
